# Platelets and inflammation—insights from platelet non-coding RNA content and release in the Bruneck study and the PACMAN-AMI trial

**DOI:** 10.1093/cvr/cvaf100

**Published:** 2025-06-03

**Authors:** Clemens Gutmann, Temo Barwari, Christian Schulte, Konstantinos Theofilatos, Bhawana Singh, Kaloyan Takov, Gonca Suna, Melissa V Chan, Paul C Armstrong, Christian Cassel, Yasushi Ueki, Jonas D Häner, Peter Santer, Peter Willeit, Christian Hengstenberg, Lorenz Räber, Stefan Kiechl, Johann Willeit, Timothy D Warner, Manuel Mayr

**Affiliations:** Division of Cardiology, Medical University of Vienna, Vienna, Austria; King’s British Heart Foundation Centre, King’s College London, London, UK; King’s British Heart Foundation Centre, King’s College London, London, UK; King’s British Heart Foundation Centre, King’s College London, London, UK; Department of Cardiology, University Heart and Vascular Center Hamburg, University Medical Center Hamburg-Eppendorf, Hamburg, Germany; German Centre of Cardiovascular Research (DZHK), Partner Site Hamburg, Hamburg, Germany; King’s British Heart Foundation Centre, King’s College London, London, UK; National Heart and Lung Institute, Imperial College London, 86 Wood Ln, London W12 0BZ, UK; National Heart and Lung Institute, Imperial College London, 86 Wood Ln, London W12 0BZ, UK; Department of Cardiology, University Heart Center Zürich, Zürich, Switzerland; The Blizard Institute, Barts and the London School of Medicine and Dentistry, Queen Mary University of London, London, UK; The Blizard Institute, Barts and the London School of Medicine and Dentistry, Queen Mary University of London, London, UK; National Heart and Lung Institute, Imperial College London, 86 Wood Ln, London W12 0BZ, UK; Department of Cardiology, Bern University Hospital, University of Bern, Bern, Switzerland; Department of Cardiology, Bern University Hospital, University of Bern, Bern, Switzerland; Department of Laboratory Medicine, Bruneck Hospital, Bruneck, Italy; Institute of Clinical Epidemiology, Public Health, Health Economics, Medical Statistics and Informatics, Medical University of Innsbruck, Innsbruck, Austria; Ignaz Semmelweis Institute, Interuniversity Institute for Infection Research, Medical University of Vienna, Vienna, Austria; Department of Public Health and Primary Care, University of Cambridge, Cambridge, UK; Division of Cardiology, Medical University of Vienna, Vienna, Austria; Department of Cardiology, Bern University Hospital, University of Bern, Bern, Switzerland; Department of Neurology, Medical University of Innsbruck, Innsbruck, Austria; VASCage—Centre on Clinical Stroke Research, Innsbruck, Austria; Department of Neurology, Medical University of Innsbruck, Innsbruck, Austria; The Blizard Institute, Barts and the London School of Medicine and Dentistry, Queen Mary University of London, London, UK; Division of Cardiology, Medical University of Vienna, Vienna, Austria; National Heart and Lung Institute, Imperial College London, 86 Wood Ln, London W12 0BZ, UK

**Keywords:** Non-coding RNA, Platelet reactivity, Anti-platelet therapy, Cardiovascular disease, Light transmission aggregometry

## Abstract

**Aims:**

Platelets contain non-coding RNAs (ncRNAs), and their measurement may complement platelet aggregometry.

**Methods and results:**

In the community-based Bruneck study (*n* = 338), we generated platelet-rich plasma (PRP), platelet-poor plasma (PPP), and platelets. PRP was subjected to aggregometry using various agonists and processed to platelet releasates thereafter. Releasates, PPP, and platelets underwent real-time polymerase chain reactions to measure ncRNAs. Platelet ncRNA release appeared agonist-specific, dose-dependent, and inhibited by aspirin. Collagen triggered the strongest release for most ncRNAs, whereas miR-150 was hyperresponsive to ADP, and miR-21 was hyperresponsive to arachidonic acid. Comparing the dynamic range of ncRNA release to aggregation, aggregation reached a maximum at high agonist concentrations, while ncRNAs continued to rise. Cohort-wide associations showed that inflammation parameters like neutrophil counts and C-reactive protein correlated inversely with platelet aggregation and ncRNA release. Similarly, a high leucocyte-derived RNA content in isolated platelets correlated inversely with aggregation. Inverse correlations were absent in aspirin users. Through experiments on plasma-free platelet releasates and platelets, including size-exclusion chromatography, ultracentrifugation, and degradation assays, we discovered that microRNAs and YRNAs are carried by proteins and readily released, while circular-, long non-coding-, and messenger RNAs are carried by vesicles and preferentially retained. Finally, we assessed ncRNA responses to short- and long-term dual anti-platelet therapy (DAPT) in plasma from 265 patients with acute myocardial infarction (AMI) of the PACMAN-AMI trial. Most of the DAPT effect was already achieved by 4 weeks, with a further reduction at 52 weeks, revealing a short- and long-term DAPT effect not captured by aggregometry.

**Conclusion:**

Inflammation and leucocyte-derived RNAs in isolated platelets are associated with reduced platelet responses *ex vivo,* potentially reflecting exhaustion through pre-activation *in vivo*. We show that protein-bound ncRNAs are readily released from platelets, whereas vesicle-bound ncRNAs are preferentially retained. We highlight the potential of ncRNAs as biomarkers complementing aggregometry.

## Graphical Abstract

**Graphical Abstract:**
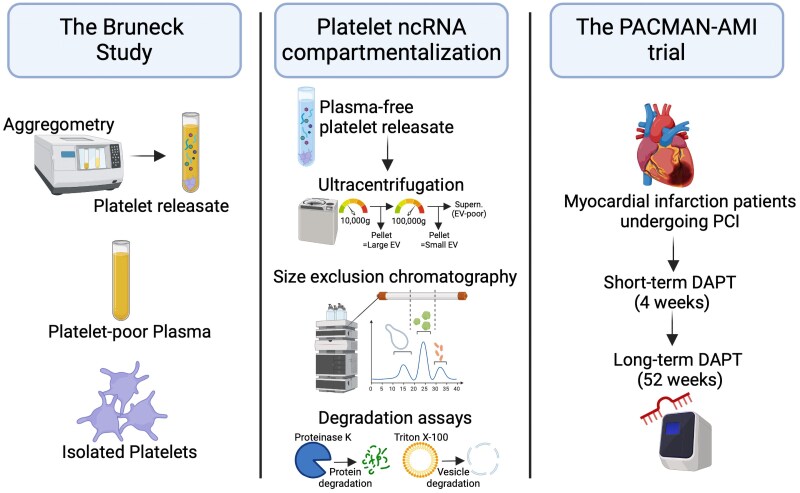

**Time of primary review: 40 days**

**See the editorial comment for this article ‘Platelet-derived RNAs: a new regulatory marker for vascular inflammation?’, by Shinya Goto *et al*., https://doi.org/10.1093/cvr/cvaf124.**

## 1. Introduction

Platelets contain various non-coding RNAs (ncRNAs), including microRNAs (miRNAs),^1–3^ YRNAs,^2–4^ long non-coding RNAs (lncRNAs),^5^ and circular RNAs (circRNAs),^6,7^ among others,^8–10^ which influence platelet function^2,11–13^. However, their role in platelet reactivity and inflammation remains unclear.^14^

In the Bruneck study, we conducted one of the most extensive assessments of *ex vivo* platelet reactivity to date.^15^ Notably, we observed an inverse association between *ex vivo* platelet aggregation and S100A8/A9, a neutrophil protein identified in platelets isolated from patients with AMI.^16^ This suggests that platelets can acquire molecular components from other cells, particularly neutrophils,^17–21^ a process exacerbated during inflammation,^19–21^ which is linked to pro-thrombotic effects.^22^

Our objective was to examine platelet ncRNA classes in the Bruneck study (*n* = 338 participants), analysing platelet aggregation via light transmission aggregometry (LTA) and measuring ncRNAs in platelet-poor plasma (PPP), platelet releasates generated during LTA, and isolated platelets. This allowed us to explore associations between platelet responses, ncRNA release, and inflammation. We then assessed changes in ncRNAs and VerifyNow aggregometry measurements with dual anti-platelet therapy (DAPT) in acute myocardial infarction (AMI) patients, using the PACMAN-AMI trial (*n* = 265 patients at 4 weeks and 52 weeks post-AMI).

## 2. Methods

Detailed methods are provided in the Supplementary material online. Sample collections in the Bruneck study, the PACMAN-AMI trial, and from healthy volunteers complied with the Declaration of Helsinki and were approved by the responsible institutional ethics committees, as described in the Supplementary material online.

### 2.1 Sample collection in the Bruneck study

**Figure 1:**
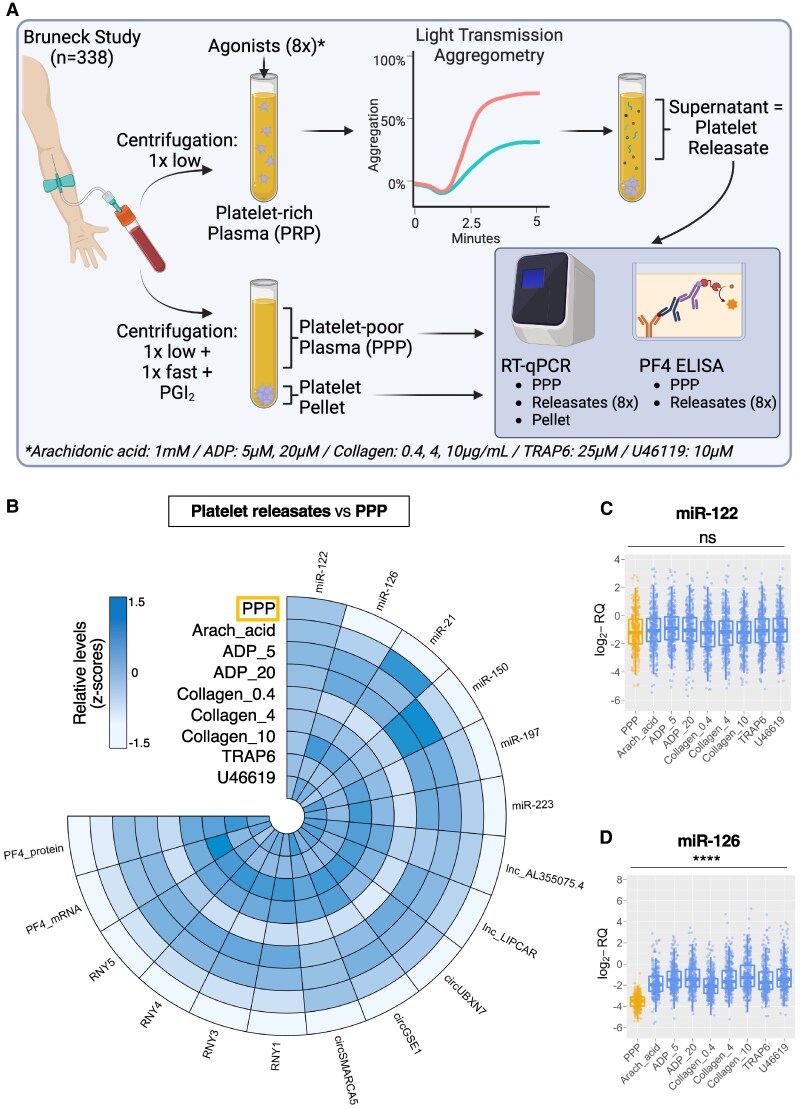
Novel and conventional platelet function measurements in the Bruneck study. (*A*) Schematic describing the measurements performed in all 338 participants of the sixth quinquennial examination of the community-based prospective Bruneck study. (*B*) Circular heatmap displaying RNA levels and PF4 protein levels in PPP and eight different platelet releasates sampled from all 338 Bruneck participants. (*C* and *D*) Representative box and whisker plots for hepatocyte-derived miR-122 (*C*) and platelet-derived miR-126 (*D*) in all 338 Bruneck participants, using Kruskal–Wallis tests for statistical comparisons. The middle line represents the median, the upper and lower box borders represent the IQR and the whiskers represent 1.5 times the IQR of log_2_-RQ RNA levels.

The Bruneck study is a longitudinal study originally started in 1990, where a sex- and age-stratified random sample of 1000 inhabitants of Bruneck (Italy) was included. In the 2015 follow-up investigation, blood of all 338 surviving participants was collected after an overnight fast and smoking abstinence. Platelet-rich plasma (PRP) was generated by centrifugation at 200 *g* for 15 min without brake, carefully aspirating only the uppermost part of the supernatant to prevent disturbance or aspiration of the buffy coat; 2 μg/mL prostacyclin was then added to prevent platelet activation. PPP (supernatants) and platelets (pellets) were obtained by centrifugation at 2200 *g* for 10 min. PRP was used for LTA for 5 min with different platelet agonists for 5 min (*Figure 1A*, Supplementary material online, *Table S1*). For LTA we used an 8-channel Bio/Data PAP-8E machine, allowing simultaneous measurement of all 8 agonist-treated samples from the same patient. Final aggregation (%) was used as the LTA readout.^15^ Aspirin use was determined using an established LTA threshold (final aggregation <20% in response to arachidonic acid) that is superior to self-report in elderly people, as described previously.^15^ Immediately after LTA, 10 U/mL heparin and 1 mM diclofenac were added to all PRP-containing cuvettes to stop aggregation and to prevent the formation of fibrin clots. We chose 1 mM diclofenac because at this concentration cyclooxygenases are completely inhibited, thereby preventing thromboxane A2 generation and any additional aggregation.^23^ Platelet releasates (supernatants) were then obtained by centrifugation at 2200 *g* for 10 min. To evaluate leucocyte contamination, we quantified leucocytes in healthy donor PPP and PRP using the ‘low white blood cell mode’ of a Sysmex XN-350 cell counter.

### 2.2 Platelet lncRNA screening by RNA-seq

RNA-Seq was performed on platelets from four healthy volunteers. LncRNAs were ranked according to abundance (see Supplementary material online, *Table S2*). In addition, lncRNAs with the gene ontology term ‘platelet’ were selected to prioritize lncRNAs associated with platelets (see Supplementary material online, *Table S3*). RNA-Seq data were deposited to the GEO repository (GSE240195).

### 2.3 Platelet lncRNA validation and selection

To validate lncRNAs identified by RNA-Seq and one additional lncRNA selected from the literature (lnc_LIPCAR),^24^ we performed RT-qPCR validation in PPP, PRP and platelet pellets from four healthy volunteers, measuring: (i) lnc_LIPCAR; (ii) the 14 most abundant lncRNAs in the RNA-Seq dataset (see Supplementary material online, *Table S2*); and (iii) the 17 lncRNAs selected based on the gene ontology term ‘platelet’ (see Supplementary material online, *Table S3*). As shown in Supplementary material online, *Figure S1*, only lnc_LIPCAR (selected from the literature)^24^ and lnc_AL3550075.4 (ranked as most abundant in RNA-Seq) were consistently detected in PPP, PRP, and platelets of healthy volunteers and therefore chosen for measurement in PPP, platelet releasates, and platelets of the Bruneck study. Using detectability in platelets as the sole criterion, regardless of abundance in PPP and PRP, we selected six additional lncRNAs for measurement in platelets of the Bruneck study (see Supplementary material online, *Figure S1*).

### 2.4 Platelet circRNA validation and selection

To identify platelet circRNAs, we reviewed the literature. Ten platelet circRNAs were selected from Alhasan *et al*.^6^ and Preußer *et al*.^7^ To confirm circularity, we performed RNase R validation of RNA isolated from platelets and plasma of four healthy volunteers (see Supplementary material online, *Figure S2*). Primers and target selection criteria are provided in Supplementary material online, *Tables S4-S6*.

### 2.5 Sample collection and platelet aggregation measurements in the PACMAN-AMI trial

To evaluate plasma ncRNA responses to short-term and long-term DAPT in AMI patients, we used the PACMAN-AMI trial (effects of the PCSK9 antibody AliroCuMab on coronary Atherosclerosis in patieNts with Acute Myocardial Infarction).^25,26^ Inclusion and exclusion criteria can be found in the study protocol (NCT03067844) and in the flow chart in Supplementary material online, *Figure S3*. Blood from 265 patients was collected from the antecubital vein 24 h after hospital admission and percutaneous coronary intervention due to AMI (*n* = 85), and at week 4 (*n* = 265) and week 52 thereafter (*n* = 265). Samples taken at baseline (*n* = 265), i.e. immediately upon hospital admission and before alirocumab or placebo administration, were omitted to avoid comparability issues, because they were collected from the arterial sheath and not the antecubital vein. The first 2–4 mL of blood were discarded to avoid spontaneous platelet activation, and samples were collected in 8.2 mL citrate tubes (3.2% sodium citrate) and processed to plasma within 1 h after blood drawing through centrifugation at 3136 *g* for 7 min.^25,26^ Platelet aggregation was measured with the VerifyNow aspirin assay and the VerifyNow P2Y_12_ assay (Accumetrics Corporation), as described previously.^26^ Plasma was frozen at −80°C.

### 2.6 RNA isolation, heparinase treatment, and RT-qPCR

For isolation of total RNA, the miRNeasy Mini kit was used according to the manufacturer’s protocol.^2,27,28^ RNA was treated with heparinase to overcome the confounding effects of heparin on RT-qPCR.^2,27,28^ RT-qPCR was performed as described previously, with samples belonging to the same patient being run on the same RT and qPCR plates to minimize bias.^27,28^ For normalization of RNAs in platelet pellets, the global average of all measured RNAs was used, while exogenous *Cel-miR-39-3p* was used for all other samples, as described previously.^27,28^

### 2.7 ELISA

PF4 protein was quantified in PPP and platelet releasates using the Human CXCL4/PF4 DuoSet ELISA and the DuoSet Ancillary Reagent Kit 2 according to the manufacturer’s instructions.

### 2.8 Compartmentalization of platelet-derived ncRNAs

The compartmentalization of platelet-derived ncRNAs was assessed in healthy volunteers as described in the Supplementary material online. In brief, we removed plasma ncRNAs from platelet ncRNAs by generating plasma-free platelet releasates. We then assessed compartmentalization by pelleting extracellular vesicles (EVs) through ultracentrifugation, by performing high-performance size-exclusion chromatography (SEC), and by degrading protein or vesicle carriers within plasma-free platelet releasate using proteinase K (11.25 U/mL) or Triton X-100 (0.1%).

### 2.9 Assessment of RNA stability in plasma

To assess stability of RNAs in plasma, we incubated PPP at 37°C. RNAs were quantified using RT-qPCR, while PF4 protein was quantified by ELISA.

### 2.10 Statistical analysis

All statistical analyses were two-tailed, with *P*-values < 0.05 considered significant. Data were log_2_-transformed, and normality was assessed using Shapiro–Wilk tests. As some variables were not normally distributed, non-parametric methods were applied throughout. Mann–Whitney *U* tests and Fisher’s exact tests were used for unpaired continuous and binary variables, respectively, while Wilcoxon signed-rank tests were used for paired continuous data. Kruskal–Wallis and Friedman tests with Dunn’s *post hoc* test assessed differences in unpaired and paired multi-group comparisons. Spearman correlation was used for continuous variables and point-biserial correlation was used for continuous vs. binary variables. Correlation coefficients and *P*-values for heat map correlations are available in the Supplementary material online, *Supplementary data files*. Platelet releasate RNA and PF4 protein levels were normalized to platelet count. Schematic diagrams were created with Biorender.com.

## 3. Results

### 3.1 Platelet function measurements in the Bruneck study

The Bruneck study is a prospective survey with an age- and sex-stratified random sample of the inhabitants of Bruneck, South Tyrol, Italy (*Table 1*). For LTA (*Figure 1A*), we used the agonists arachidonic acid (AA, 1 mM), ADP (5 and 20 µM), collagen (0.4, 4, and 10 µg/mL), the PAR-1 agonist TRAP6 (25 µM), and the thromboxane receptor agonist U46119 (10 µM). These agonists and their concentrations were previously shown to elicit robust aggregation responses and enable assessment of anti-platelet therapy.^29,30^ For ADP, we chose 5 µM (sensitive) and 20 µM (maximal response), as used in the PLATO trial.^31^ For collagen, three concentrations were chosen since lower doses are aspirin-sensitive, while higher ones are not.^29,30^ Fasting blood samples from all 338 participants were immediately processed to produce PPP (*n* = 338) and platelet releasates (*n* = 2704) during LTA.

**Table 1:** Demographics of the Bruneck cohort (2015 follow-up investigation)

Demographics	Full cohort (*n* = 338)	No aspirin (*n* = 183)	Aspirin (*n* = 155)	*P*-value
Age (years)	74 (70, 79)	73 (69, 79)	76 (71, 80)	**0**.**028**
Body mass index (kg/m^2^)	25.2 (22.6, 27.8)	24.4 (22.4, 27.4)	25.9 (23.1, 28.4)	**0**.**024**
Ethnicity (Caucasian)	338 (100%)	183 (100%)	155 (100%)	0.99
Sex (males)	165 (48.8%)	92 (50.3%)	73 (47.1%)	0.59
Underlying conditions				
History of cerebrovascular disease	22 (6.5%)	11 (6.0%)	11 (7.1%)	0.83
History of myocardial infarction	16 (4.7%)	1 (0.5%)	15 (9.7%)	**<0**.**001**
Smoking	43 (12.7%)	19 (10.4%)	24 (15.5%)	0.19
Type 2 diabetes	28 (8.3%)	9 (4.9%)	19 (12.3)	**0**.**017**
Medication				
Anti-coagulation	44 (13.0%)	34 (18.6%)	10 (6.5%)	**0**.**001**
Direct oral anti-coagulants	14 (4.1%)	10 (5.5%)	4 (2.6%)	0.27
Vitamin K antagonists	30 (8.9%)	24 (13.1%)	6 (3.9%)	**0**.**003**
Lipid-lowering drugs	110 (32.5%)	45 (24.6%)	65 (41.9%)	**0**.**001**
Ezetimibe	3 (0.9%)	0 (0%)	3 (1.9%)	0.10
Statins	109 (32.2%)	45 (24.6%)	64 (41.3%)	**0**.**002**
Anti-diabetic drugs	24 (7.1%)	8 (4.4%)	16 (10.3%)	0.05
Insulin	5 (1.5%)	2 (1.1%)	3 (1.9%)	0.66
Oral anti-diabetic drugs	22 (6.5%)	6 (3.3%)	4 (2.6%)	0.76
Other drugs				
Anti-depressants	38 (11.2%)	16 (8.7%)	22 (14.2%)	0.12
Anti-hypertensive drugs	205 (60.7%)	93 (50.8%)	112 (72.3%)	**<0**.**001**
Blood pressure				
Systolic blood pressure (mm Hg)	149 (136, 162)	146 (131, 162)	151 (139, 164)	**0**.**010**
Diastolic blood pressure (mm Hg)	86 (80, 94)	86 (80, 93)	86 (80, 94)	0.72
Laboratory parameters				
CRP (mg/L)	5.0 (5.0, 5.8)	5.0 (5.0, 6.0)	5.0 (5.0, 5.3)	0.15
Creatinine (mg/dL)	0.9 (0.8, 1.0)	0.9 (0.8, 1.0)	0.9 (0.8, 1.0)	0.37
Gamma-GT (U/L)	20.0 (13.1, 32.3)	18.1 (13.1, 29.0)	22.1 (13.1, 36.0)	0.09
Glucose (mmol/L)	5.2 (4.8, 5.7)	5.2 (4.7, 5.7)	5.3 (4.9, 5.9)	**0**.**033**
HbA_1C_ (%)	5.6 (5.4, 5.8)	5.5 (5.4, 5.8)	5.6 (5.4, 5.9)	**0**.**018**
Lipids				
High density lipoprotein (mmol/L)	1.59 (1.33, 1.86)	1.63 (1.37, 1.93)	1.53 (1.30, 1.82)	**0**.**036**
Low density lipoprotein (mmol/L)	3.29 (2.59, 4.04)	3.50 (2.75, 4.15)	3.08 (2.46, 3.71)	**0**.**001**
Total cholesterol (mmol/L)	5.18 (4.47, 5.86)	5.36 (4.62, 6.01)	4.85 (4.30, 5.48)	**<0**.**001**
Triglycerides (mg/dL)	93 (74, 121)	92 (74, 119)	94 (72, 123)	0.79
Blood count				
Hematocrit (%)	41 (39, 44)	41 (39, 44)	41 (40, 44)	0.63
Haemoglobin (g/L)	140 (134, 150)	140 (134, 150)	141 (134, 149)	0.75
Leucocytes (10^9^/L)	5.7 (4.9, 6.7)	5.6 (4.7, 6.7)	5.9 (5.1, 6.8)	0.16
Platelet count (10^9^/L)	217 (182, 261)	219 (188, 259)	213 (177, 267)	0.45
Mean platelet volume (fL)	10.5 (9.9, 11.0)	10.5 (9.9, 11.1)	10.4 (10.0, 11.0)	0.94
Red blood cells (10^12^/L)	4.62 (4.39, 4.92)	4.64 (4.39, 4.92)	4.58 (4.39, 4.92)	0.68
Neutrophils (10^9^/L)	3.38 (2.60, 4.09)	3.20 (2.50, 4.04)	3.40 (2.75, 4.09)	0.15
Neutrophils (% of leucocytes)	57.3 (52.4, 63.8)	57 (51.9, 61.8)	58.6 (53.1, 65.0)	0.13
Lymphocytes (10^9^/L)	1.7 (1.3, 2.1)	1.7 (1.4, 2.2)	1.6 (1.3, 2.0)	0.24
Monocytes (10^9^/L)	0.5 (0.4, 0.6)	0.5 (0.4, 0.6)	0.5 (0.4, 0.6)	0.25

For continuous variables, medians and inter-quartile ranges are shown, and Mann–Whitney *U* tests were performed to determine the significance between participants not on aspirin and participants on aspirin. For binary variables, absolute counts and percentages are shown, and Fisher’s exact tests were performed to determine statistical significance. *P*-values below 0.05 are highlighted in bold font. Aspirin use was determined using an established LTA threshold (final aggregation < 20% to AA), as previously described.^15^

### 3.2 Platelet ncRNA measurements in the Bruneck study

We selected representative ncRNAs from four RNA classes (see Supplementary material online, *Table S4*): MiRNAs and YRNAs were chosen based on previous RNA-Seq results.^2^ For lncRNAs, we conducted an RNA-Seq experiment in platelets and selected 14 lncRNAs based on abundance (see Supplementary material online, *Table S2*) and 17 with the gene ontology term ‘platelet’ (see Supplementary material online, *Table S3*). Lnc_AL3550075.4 was the most abundant in the RNA-Seq data and consistently detected by RT-qPCR (see Supplementary material online, *Figure S1*). We included mitochondrial-derived lnc_LIPCAR due to its association with cardiovascular outcomes.^24^ For circRNAs, we selected three (circUBXN7, circGSE1, and circSMARCA5) from previous studies^6,7^ and confirmed their circularity by RNase R experiments (see Supplementary material online, *Figure S2*). We also assessed PF4 mRNA and protein levels, which correlate with platelet miRNAs and YRNAs.^2^ All RNAs and PF4 were measured in PPP (*n* = 338) and in eight platelet releasates to different agonists (*n* = 2704).

### 3.3 Comparison of ncRNA levels in platelet releasates to PPP in the Bruneck study

*Figure 1B* presents a circular heat map displaying selected miRNAs, lncRNAs, circRNAs as well as PF4 mRNA and protein levels in samples from the Bruneck study. Average expression levels in the different releasates were compared to PPP. As expected, levels of liver-derived miR-122^32^ in the platelet releasates were similar to those in PPP (*Figure 1C*). In contrast, platelet releasates showed a dose-dependent increase of platelet ncRNAs, along with PF4 protein and mRNA, compared to PPP. Endothelial- and platelet-enriched miR-126 is shown as an example in *Figure 1D*. Individual box plots for all other measurements are shown in Supplementary material online, *Figure S4*.

### 3.4 Platelet ncRNA release is agonist-specific and inhibited by aspirin in the Bruneck study

**Figure 2:**
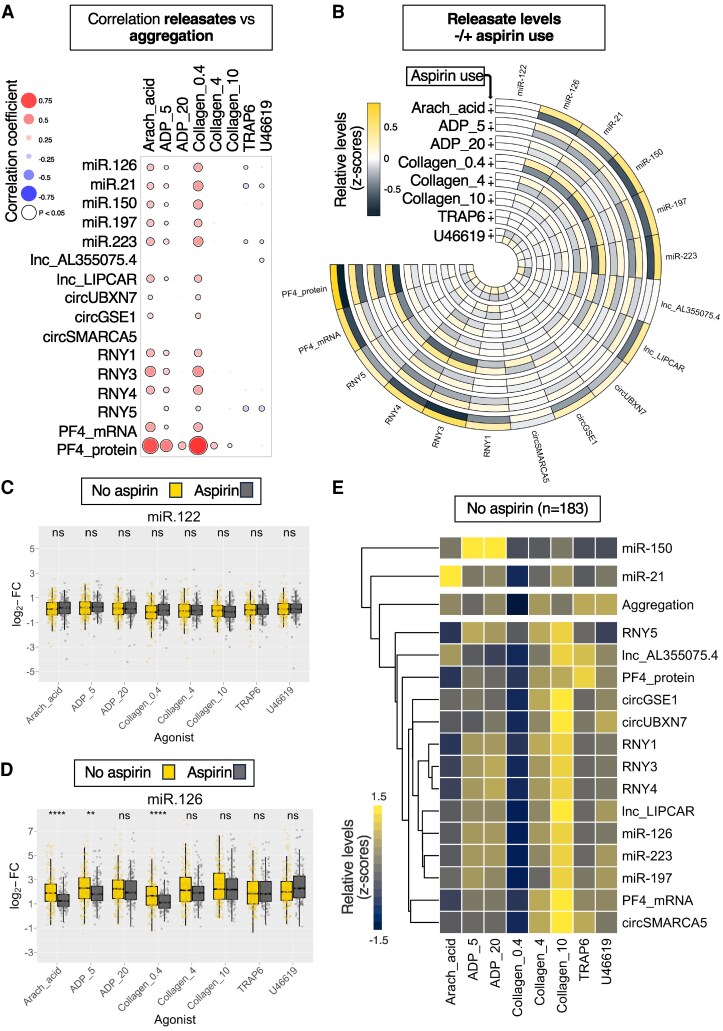
*Ex vivo* RNA and protein release is agonist-specific and inhibited by aspirin in the Bruneck study. (*A*) Spearman correlation between aggregation responses and releasate RNA and PF4 protein levels as fold changes (FC) of platelet releasate levels to PPP levels in all 338 Bruneck participants. The size and colour intensity of dots reflect correlation strength. Blue reflects inverse and red reflects positive correlations. Significant (*P* < 0.05) correlations are highlighted by a black circle around the dot. (*B*) Circular heatmap displaying RNA levels and PF4 protein levels as FC of platelet releasate levels to PPP levels, comparing participants not on aspirin (‘−’, *n* = 183) to participants on aspirin (‘+’, *n* = 155). (*C* and *D*) Representative box and whisker plots for hepatocyte-derived miR-122 (*C*) and platelet-derived miR-126 (*D*), comparing participants not on aspirin (*n* = 183) to participants on aspirin (*n* = 155) using Mann–Whitney *U* tests in each agonist group. The middle line represents the median, the upper and lower box borders represent the IQR and the whiskers represent 1.5 times the IQR of log_2_-FC RNA levels. (*E*) Hierarchically clustered heatmap displaying *ex vivo* RNA and PF4 protein release as FC of releasate levels to PPP levels, as well as *ex vivo* aggregation responses in participants not on aspirin (*n* = 183).

We explored correlations between *ex vivo* aggregation, assessed via LTA, and ncRNA release following agonist stimulation. The release of ncRNAs correlated with aggregation responses to AA and lower ADP and collagen concentrations (*Figure 2A*), but not with higher ADP and collagen concentrations or potent agonists (TRAP6 and U46619). A similar pattern was observed for PF4 protein, with stronger correlations at lower agonist concentrations. For instance, PF4 protein showed a strong correlation at collagen 0.4 µg/mL (*r* = 0.80, *P* < 0.0001) but a weaker correlation at collagen 10 µg/mL (*r* = 0.13, *P* = 0.02). Most released ncRNAs strongly correlated with each other, while PF4 protein clustered separately with weaker correlations to ncRNAs (see Supplementary material online, *Figure S5*). Correlations between PF4 protein and ncRNAs were stronger with weaker agonists and diminished with potent agonist stimulation (see Supplementary material online, *Figure S5*).

We next investigated the impact of aspirin on *ex vivo* platelet ncRNA release. As shown in *Figure 2B*, aspirin users (*n* = 155) exhibited reduced platelet ncRNA release compared to non-aspirin users (*n* = 183), while liver-derived miR-122 levels remained unchanged. Representative box plots for miR-122 (*Figure 2C*) and miR-126 (*Figure 2D*) are shown, with all other measurements, including PF4 mRNA/protein, in Supplementary material online, *Figure S6*. Aspirin’s inhibitory effect was most pronounced with AA and weaker agonists such as ADP and low collagen (0.4 and 4 µg/mL) but had no impact on stimulation by high collagen (10 µg/mL), TRAP6, and U46119. Anti-coagulants had no effect on aggregation or ncRNA release (data not shown).

To assess variations in ncRNA release across different agonists, we compared responses in aspirin-naive participants (*n* = 183, *Figure 2E*). Fold-change comparisons for all agonists are shown in Supplementary material online, *Figure S7*. High-dose collagen induced the strongest release of most ncRNAs and was a more potent stimulus for ncRNA release than platelet aggregation or PF4 protein release (*Figure 2E* and Supplementary material online, *Figure S7*). In contrast, TRAP6 was the most potent stimulus for PF4 protein release (*Figure 2E* and Supplementary material online, *Figure S7*). Notably, miR-150 exhibited a heightened response to ADP, while miR-21 was hyperresponsive to AA (*Figure 2E* and Supplementary material online, *Figure S7*). This exaggerated miR-21 response to AA was absent in aspirin users (*n* = 155) (see Supplementary material online, *Figure S8*).

### 3.5 Correlation of platelet aggregation with clinical variables in the Bruneck study

**Figure 3:**
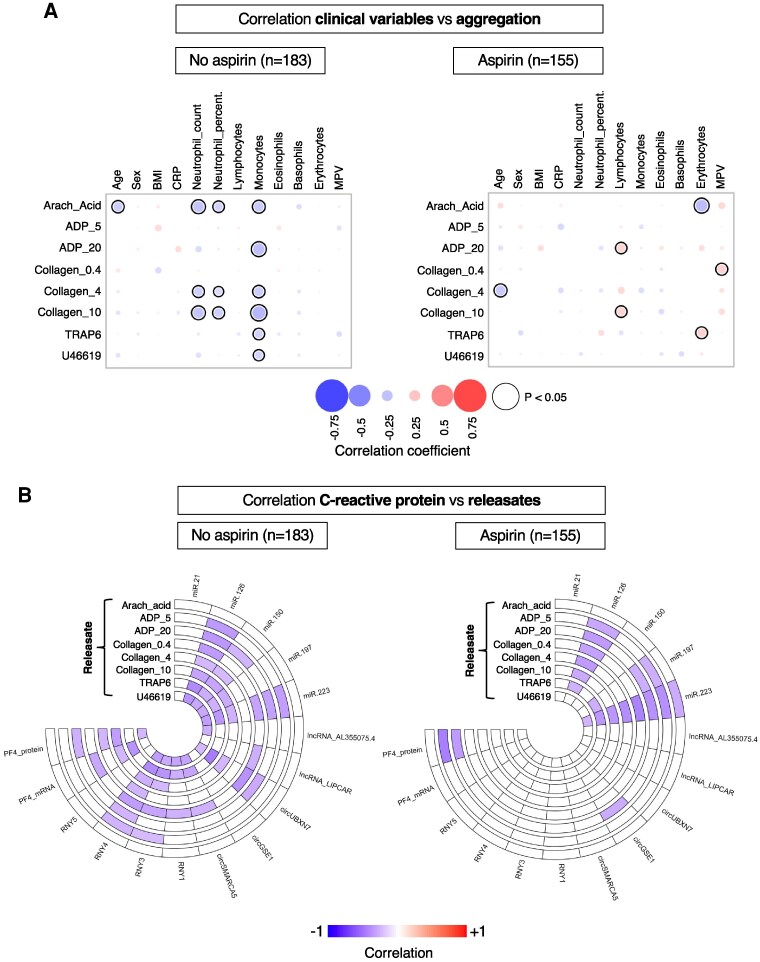
Platelet aggregation and release measurements reveal platelet exhaustion in inflammation in the Bruneck study. (*A*) Correlation between clinical variables and aggregation responses in participants not on aspirin (left panel, *n* = 183) and participants on aspirin (right panel, *n* = 155). Size and colour intensity of dots reflect correlation strength. Blue reflects inverse and red reflects positive correlations. Significant (*P* < 0.05) correlations are highlighted by a black circle around the dot. Correlations between continuous variables are based on Spearman correlation. Correlations between a continuous and a binary variable are based on Point-biserial correlation. (*B*) Circular heatmaps displaying Spearman correlations between CRP and releasate RNA and PF4 protein levels in non-aspirin users (left panel, *n* = 183) and aspirin users (right panel, *n* = 155). Correlations with *P* ≥ 0.05 are uncoloured.

Next, we investigated associations between clinical variables and aggregation, focusing on inflammatory parameters such as leucocyte counts (including neutrophils and monocytes) and C-reactive protein (CRP) levels. In aspirin-naive participants (*n* = 183), aggregation responses to multiple agonists inversely correlated with neutrophil counts, neutrophil-to-leucocyte ratio, and monocyte counts (*Figure 3A*, left panel). However, these correlations were absent in aspirin users (*n* = 155) (*Figure 3A*, right panel). Thus, circulating inflammatory cells were linked to reduced *ex vivo* platelet responses in LTA measurements among aspirin-naive individuals, but this correlation was diminished in aspirin users.

### 3.6 Correlation of ncRNA release with markers of inflammation in the Bruneck study

We then investigated the correlations between CRP (*Figure 3B*), and other inflammation markers (see Supplementary material online, *Figure S9*), with platelet ncRNAs following agonist stimulation. CRP levels and leucocyte counts did not differ significantly between aspirin users and non-users (*Table 1*). Among aspirin-naive participants, CRP levels exhibited inverse associations with several platelet-derived ncRNAs (*Figure 3B*, left panel and Supplementary material online, *Figure S9*). In aspirin users (*n* = 155), only few inverse correlations with CRP persisted, specifically miR-126, miR-197, and miR-223 (*Figure 3B*, right panel and Supplementary material online, *Figure S9*). We have previously linked these three miRNAs to the risk of AMI and cardiovascular death.^33^ No significant correlations were observed with leucocyte counts in either aspirin users or non-users (see Supplementary material online, *Figure S9*). These findings suggest that elevated levels of CRP are associated with decreased levels of platelet ncRNA release upon *ex vivo* stimulation, indicating potential ‘exhaustion’ of platelets during sub-clinical inflammation. This effect is attenuated by aspirin and prompted us to further investigate the possibility of horizontal RNA transfer from leucocytes to platelets.

### 3.7 Expression profiles of RNAs in isolated platelets in the Bruneck study

Platelets were isolated from all 338 participants during the same blood donation (*Figure 1A*). In order to assess the potential horizontal RNA transfer from leucocytes to platelets,^16^ we measured mRNA levels of leucocyte markers (PTPRC, S100A8, and S100A9) and platelet markers (PF4, PPBP, and ITGA2B). The relatively high RNA levels in platelets, compared to PPP and platelet releasates, allowed for detection of less abundant RNAs. We expanded our panel of RNAs (see Supplementary material online, *Table S4*) to include the complete list of platelet circRNAs validated in our pilot experiment (see Supplementary material online, *Figure S2*), and their less abundant linear isoforms.^6,7^ In addition, we included six additional lncRNAs (lncRNAs RMRP, LINC02284, AL954642.1, AC147067.1, LINC00989, and AC026785.2) identified from our platelet RNA-Seq data and detectable in healthy volunteers by RT-qPCR (see Supplementary material online, *Figure S1*).

**Figure 4:**
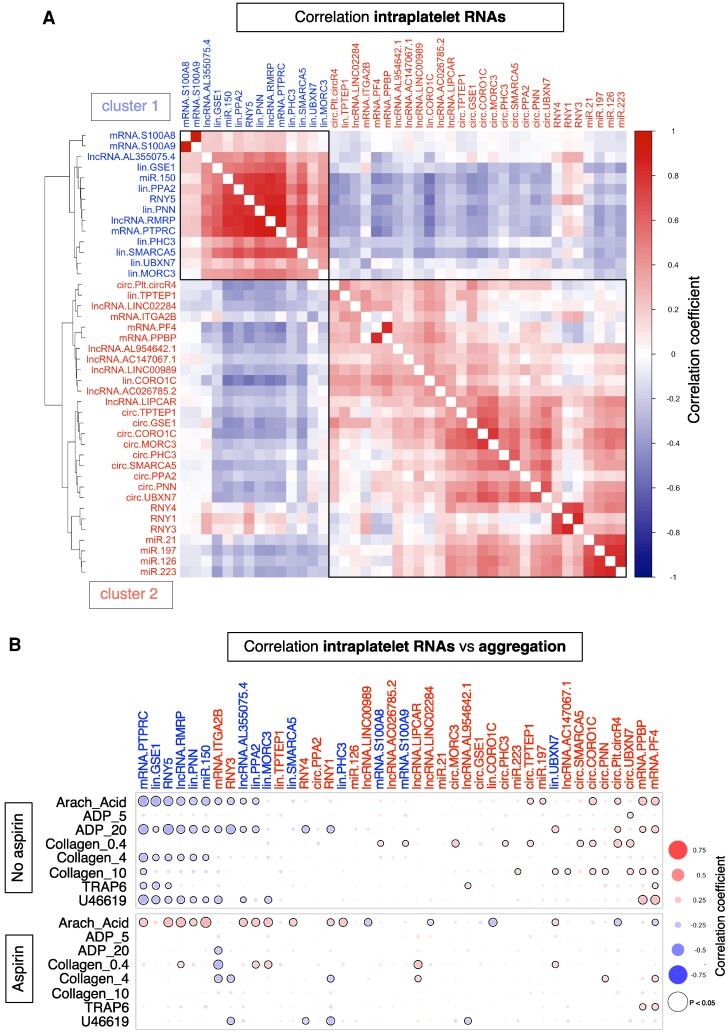
Intra-platelet RNA levels and their association with aggregation responses in the Bruneck study. (*A*) Hierarchical cluster analysis on a Spearman correlation heatmap of intra-platelet RNAs measured in all 338 Bruneck participants. Cluster one is labelled in blue and highlights RNAs that cluster with known leucocyte markers such as PTPRC and S100A8/A9 mRNAs. Cluster two is labelled in red and highlights RNAs that cluster with known platelet markers such as PF4 and PPBP mRNAs. (*B*) Spearman correlations between intra-platelet RNA levels and platelet aggregation responses in participants not on aspirin (upper panel, *n* = 183) and participants on aspirin (lower panel, *n* = 155). Size and colour intensity of dots reflect correlation strength. Blue reflects inverse and red reflects positive correlations. Significant (*P* < 0.05) correlations are highlighted by a black circle around the dot. Intra-platelet RNA labels are coloured according to their clustering in panel (*A*), i.e. leucocyte-derived RNA cluster (cluster one, blue font) or platelet RNA cluster (cluster two, red font). RNAs in panel (*B*) are ranked from left to right from the most negative to the most positive average correlation coefficient across all agonists in non-aspirin users.

Consistent with previous reports,^6,7^ intra-platelet circRNAs displayed higher abundance compared to their respective linear isoforms. Nonetheless, all linear isoforms, except for linPlt-circR4, remained detectable (see Supplementary material online, *Figure S10*). Hierarchical cluster analysis of a Spearmans correlation heatmap revealed two distinct clusters (*Figure 4A*):

1. The first cluster encompassed leucocyte-associated RNAs, including S100A8/A9 and PTPRC (CD45); miR-150; RNY5; and two lncRNAs that were selected from our RNA-Seq data based on abundance (*i.e.* lnc_AL355075.4 and lnc_RMRP). This ‘leucocyte RNA cluster’ contained most linear isoforms of circRNAs.
2. The second cluster comprised platelet markers such as mRNAs for PF4, platelet pro-basic protein (PPBP), and integrin alpha-2B (ITGA2B). This cluster also included most miRNAs in addition to RNY1, 3, and 4 and all other lncRNAs selected based on the gene ontology term ‘platelet’ or from the literature, i.e. lnc_LIPCAR. All circRNAs were associated with this platelet cluster, reinforcing the notion of circRNA enrichment in platelets.^6,7^ However, only the linear isoforms of two circRNAs (linTPTEP1, linCORO1C) were part of the same cluster, indicating a higher degree of platelet specificity for their lin/circRNA ratios.

### 3.8 Association of intra-platelet RNAs with aggregation in the Bruneck study

Next, we investigated associations between platelet RNA levels and platelet aggregation to determine whether the presence of leucocyte-derived RNAs in isolated platelets is linked to impaired platelet function. In aspirin-naive participants (*n* = 183), several RNAs from the leucocyte cluster (cluster one, *Figure 4A*) displayed inverse correlations with aggregation responses (*Figure 4B*, lower panel). Conversely, distinct intra-platelet RNAs from the platelet cluster (cluster two, *Figure 4A*) exhibited positive correlations with aggregation (*Figure 4B*, upper panel). Thus, the presence of leucocyte-derived RNAs in isolated platelets was associated with refractory platelet aggregation following *ex vivo* stimulation. Notably, in aspirin users (*n* = 155), these patterns were absent.

### 3.9 Compartmentalization of RNAs released from platelets assessed in samples from healthy donors

**Figure 5:**
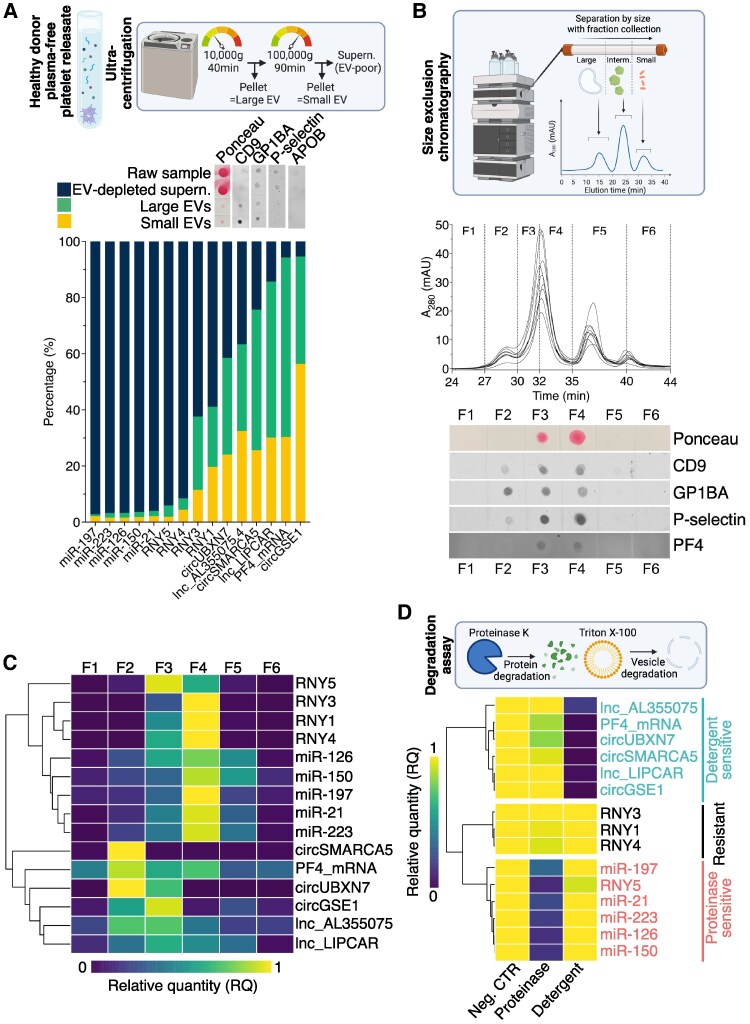
Platelet-derived miRNAs and YRNAs are predominantly carried by proteins, while circRNAs, lncRNAs and mRNA are carried by vesicles in samples from healthy donors. (*A*) The upper panel displays a schematic of the ultracentrifugation experiment conducted on plasma-free platelet releasate. The middle panel shows the relative distribution of protein markers using dot blots on fractions of small EVs, large EVs and EV-poor supernatant generated by ultracentrifugation. The lower panel displays the relative distribution of RNAs in the same fractions. Ultracentrifugation was performed on four sample pools, with each pool containing samples from six healthy donors. (*B*) The upper panel displays a schematic of the size-exclusion chromatography (SEC) experiment conducted on plasma-free platelet releasate (*n* = 9). The middle panel displays the SEC chromatogram and shows how the six SEC fractions (F1-F6) were collected. The lower panel displays the relative distribution of protein markers in vesicle-rich early fractions and protein-rich late fractions using dot blots. (*C*) Distribution of RNAs in the SEC fractions described in panel (*B*). (*D*) The upper panel displays a schematic of the degradation assay conducted on plasma-free platelet releasate (*n* = 6). The lower panel displays relative RNA levels in samples treated with Tyrode’s HEPES buffer (negative control), proteinase K or Triton X-100. Corresponding bar graphs with statistical analyses are found in Supplementary material online, *Figure S11*.

To better understand the agonist-induced ncRNA release from platelets, we focused on exploring ncRNA carriers, including proteins and EVs. Platelets from healthy donors were resuspended in Tyrode’s HEPES buffer and stimulated *ex vivo* to generate platelet releasates, removing any plasma background. We then employed three distinct methodologies:

1. Ultracentrifugation: plasma-free platelet releasates were separated into large EVs, small EVs and EV-depleted supernatants (*Figure 5A*, upper panel). Separation validation was done by dot blots (*Figure 5A*, middle panel). The EV fractions contained predominantly circRNAs, lncRNAs and PF4 mRNA (*Figure 5A*, lower panel), while miRNAs and YRNAs were detected in EV-depleted supernatants.
2. SEC: EVs and proteins were segregated based on their sizes (*Figure 5B*, upper and middle panel). Dot blots validated the enrichment of EV markers in early fractions, while the alpha-granule protein PF4 appeared exclusively in protein-rich fractions (*Figure 5B*, lower panel). Consistent with ultracentrifugation, circRNAs, lncRNAs, and PF4 mRNA eluted in the EV-containing fractions (*Figure 5C*), while miRNAs and YRNAs emerged in the protein-rich fractions.
3. Degradation assays: Plasma-free platelet releasates were treated with proteinase (proteinase K) or detergent (Triton X-100) to selectively degrade proteins or EVs, respectively.^34^ Proteinase selectively degraded all miRNAs and RNY5, yet exhibited no effect on circRNAs, lncRNAs, or PF4 mRNA (*Figure 5D* and Supplementary material online, *Figure S11*). Conversely, detergent selectively degraded all circRNAs, lncRNAs, and PF4 mRNA, while leaving miRNAs and RNY5 unaffected. Intriguingly, all YRNAs, apart for RNY5, demonstrated resistance to both detergent and proteinase (*Figure 5D* and Supplementary material online, *Figure S11*).

Thus, all three approaches corroborated the distinct compartmentalization of circRNAs and lncRNAs within EVs, and miRNAs and YRNAs within protein-rich fractions.

### 3.10 Impact of differential compartmentalization on ncRNA release in healthy donors

**Figure 6:**
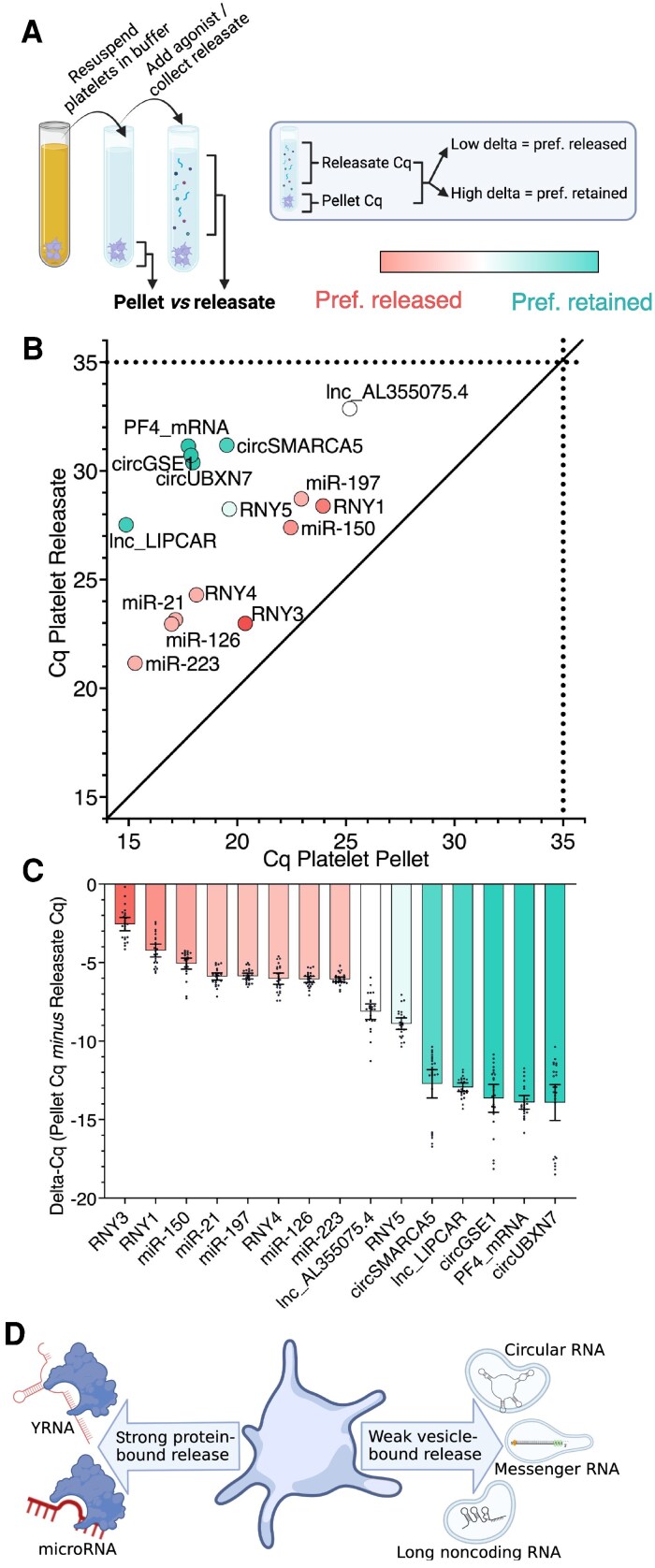
Platelet-derived miRNAs and YRNAs are readily released, while circRNAs, lncRNAs and mRNA are preferentially retained in samples from healthy donors. (*A*) Schematic describing the design of the experiments conducted on paired plasma-free platelet releasate and platelet pellet samples, to assess preferential release and preferential retainment of platelet RNAs (*n* = 24). (*B*) Cq levels of RNAs measured in plasma-free platelet releasate (y-axis) and paired, not activated platelet pellets (x-axis, *n* = 24). (*C*) Delta-Cq values calculated by subtracting the Cq value of plasma-free platelet releasate from the Cq value of the platelet pellet from the same donor. RNAs are coloured from low delta-Cq (preferentially released, red font) to high delta-Cq (preferentially retained, turquoise font). Bar graphs display means with 95% CI and individual values (*n* = 24). (*D*) Visual summary of compartmentalization and release tendency of platelet-derived RNAs.

To examine the impact of differential compartmentalization on ncRNA release, we conducted pairwise RNA measurements in plasma-free platelet releasate and unstimulated platelets (*Figure 6A*). Plotting the Cq values of RNAs in platelet pellets against Cq values in plasma-free platelet releasates revealed distinct release patterns. Notably, lnc_LIPCAR and miR-223 were among the most abundant intra-platelet RNAs (*Figure 6B*, with the lowest Cq values on the x-axis), but exhibited significant differences in their abundance within releasates (*Figure 6B*, y-axis). Overall, miRNAs and YRNAs showed a greater propensity for release (*Figure 6C*), while circRNAs, lncRNAs, and PF4 mRNA were preferentially retained. This observation aligns with our finding that circRNAs, lncRNAs and mRNAs are carried by EVs, while platelet-derived miRNAs and YRNAs are predominantly associated with proteins (*Figure 6D*).

To exclude the possibility that the observed preferential retention of circRNAs and lncRNAs is due to increased extracellular degradation, we incubated plasma samples at 37°C for various time intervals to assess extracellular ncRNA stability. As shown in Supplementary material online, *Figure S12*, all four ncRNA classes displayed a reduction in abundance levels over time, particularly when compared to PF4 protein. However, at early time points, levels of most circRNAs and lncRNAs exhibited greater stability than small ncRNAs. Thus, we can rule out faster degradation.

### 3.11 Responsiveness of circulating ncRNA levels to anti-platelet therapy in the PACMAN-AMI trial

**Figure 7:**
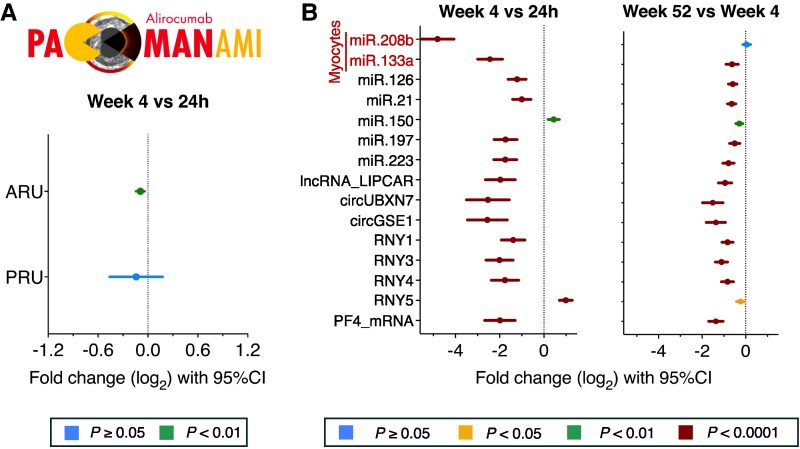
Platelet function measurements in the PACMAN-AMI trial. (*A*) Platelet aggregation was measured with the VerifyNow aspirin assay (*n* = 126 paired samples) and the VerifyNow P2Y_12_ assay (*n* = 131 paired samples). Forest plots show log_2_ fold changes with 95% confidence intervals of platelet aggregation measured as aspirin reaction units (ARU) and P2Y_12_ reaction units (PRU). (*B*) NcRNA levels were measured by RT-qPCR in 265 patients of the PACMAN-AMI trial at 24 h (*n* = 85), 4 weeks (*n* = 265) and 52 weeks (*n* = 265) post-hospital admission and percutaneous coronary intervention due to AMI. MiR-208b-3p and miR-133a are myocyte-derived, miR-150 and RNY5 are linked to inflammation, all other ncRNAs are platelet-derived. Forest plots show log_2_ fold changes with 95% confidence intervals of ncRNAs between week 4 and 24 h (*n* = 85 paired samples, left panel) and between week 52 and week 4 (*n* = 265 paired samples, right panel). Wilcoxon signed-rank test was applied in panel (*A*) and (*B*) to determine statistical significance. *P*-values are colour-coded.

In the PACMAN-AMI trial, all patients were administered statins and randomized to receive either a placebo or alirocumab followed by detailed coronary artery disease assessment over a 1-year follow-up period using imaging techniques.^25^ In our previous study,^26^ the additional LDL-lowering by alirocumab had no effect on either conventional platelet aggregation or ncRNA levels. Similarly, we did not detect significant correlations between statin use and aggregation or ncRNA release in the Bruneck study (data not shown). Due to the immediate effect of DAPT on platelet aggregation, aggregometry measurements cannot capture its short- and long-term effects (*Figure 7A*).^26^ To assess the effect of DAPT on longitudinal changes in ncRNAs post-AMI, we measured key platelet-related ncRNAs at 24 h (*n* = 85), 4 weeks (*n* = 265), and 52 weeks post-AMI (*n* = 265). As expected, cardiac miRNAs^27^ significantly decreased after AMI (*Figure 7B*). Similarly, there was a marked reduction in all platelet-related ncRNAs, except miR-150 and RNY-5, which are linked to inflammation. Even at 52 weeks, platelet ncRNAs decreased further compared to 4 weeks post-AMI.

## 4. Discussion

Clinical trials aimed at personalizing anti-platelet therapy to individual platelet reactivity have relied on *ex vivo* aggregation but have failed to show clear benefits.^35–39^ Our study highlights the agonist-specific nature of platelet ncRNA release and its susceptibility to inhibition by anti-platelet therapy. Notably, miR-150 was hyperresponsive to ADP, and miR-21 was hyperresponsive to AA. These findings align with our previous research, where aspirin (inhibiting the AA pathway) or P2Y_12_ inhibitors (inhibiting the ADP pathway) were studied for their effects on miRNA levels in healthy volunteers and patients with symptomatic carotid atherosclerosis.^1^ P2Y_12_ inhibition led to decreased miR-150 levels, while aspirin did not.^1^ Conversely, aspirin reduced miR-21 levels in a dose-dependent fashion, while P2Y_12_ inhibitors had no effect.^1^ The increased release of miR-150 to ADP and miR-21 to AA is intriguing, given the literature on miR-150, ADP and inflammation. MiR-150 is contained in both platelets and leucocytes^40^ and was assigned to the ‘leucocyte cluster’ of RNAs in isolated platelets. ADP plays an important role in platelet-dependent leucocyte activation.^41–43^ Thus, the hyper-response of miR-150 to the ADP/P2Y_12_ pathway may reflect platelet-leucocyte inter-actions.

Our study reveals inverse correlations between inflammatory markers and platelet reactivity upon agonist stimulation. Existing literature describes platelet exhaustion *ex vivo* due to *in vivo* pre-activation,^44–53^ with evidence from studies on patients with COVID-19^44^ and allergic asthma.^45^ In trauma patients,^46–50^ platelet exhaustion has been linked to increased catecholamine signaling.^50^ Historical studies from as early as the 1970s also report refractory LTA responses to agonist stimulation during surgery^51^ and in rabbit models.^52,53^ In the Bruneck study, we previously observed inverse associations between leucocyte-derived S100A8/A9 plasma levels and *ex vivo* platelet reactivity.^16^ These associations were absent in aspirin users, despite unchanged CRP levels, as low-dose aspirin does not affect high-sensitive CRP levels.^54^ Aspirin also did not alter neutrophil and monocyte counts, though it may influence unmeasured inflammatory mediators affecting platelets, such as platelet-leucocyte inter-actions.^55^ While platelet exhaustion may explain our findings, further mechanistic studies are needed, as correlations do not imply causality.

It is increasingly evident that platelets inherit a primary transcriptome from megakaryocytes and acquire a secondary transcriptome through horizontal transfer.^17,19–21,56,57^ The latter process involves the transfer of molecules from other cells, such as neutrophils, to platelets, occurring both in the vasculature^19^ and within bone marrow megakaryocytes, a phenomenon known as emperipolesis.^20,21^ Notably, we previously observed neutrophil-derived S100A8/A9 uptake by platelets.^16^ This neutrophil-to-platelet transfer is exacerbated in inflammation.^19–21^ Supporting this concept, recent RNA-Seq data have revealed small ncRNAs that are enriched in platelets but are scarce in megakaryocytes, suggesting uptake from non-megakaryocyte cells.^10^ Horizontal RNA transfer may explain why smaller platelets, a surrogate for older platelets, harbour a more diverse set of transcripts than larger platelets, which are typically considered younger platelets and contain more RNA.^18^ Our objective was to differentiate between primary and secondary platelet transcriptome by measuring platelet and leucocyte mRNA markers alongside ncRNAs. We identified two distinct RNA clusters: one centred on leucocyte markers and another on platelet markers. Notably, platelet pellet RNAs from the leucocyte cluster inversely correlated with aggregation responses, suggesting that increased leucocyte-to-platelet transfer^19–21^ is associated with platelet exhaustion. This effect was only observed in aspirin-naive participants. Although leucocytes were undetectable in PPP and low in PRP (20–30 leucocytes and 300 000 platelets per microliter PRP), we cannot rule out that some leucocyte transcripts in platelet pellets originate from co-isolated leucocytes rather than *in vivo* horizontal transfer. However, the biological implications remain: increased platelet-leucocyte inter-actions inversely associate with *ex vivo* platelet reactivity in aspirin-naive participants but not in aspirin users.

To complement insights from the Bruneck study, we explored ncRNA compartmentalization and release mechanisms. Previous investigations primarily focused on ncRNAs within the same class without examining inter-class differences. Studies,^58,59^ including our own,^1,33,60^ have proposed miRNA encapsulation within EVs in plasma and serum. As miRNAs are abundant in plasma, any contamination of EV preparations with plasma miRNAs is readily detected by RT-qPCR. In this study, we found limited release of platelet-derived miRNAs and YRNAs within EVs, suggesting their low copy numbers within EVs are likely insufficient to mediate substantial biological effects.^61^ Conversely, lncRNAs and circRNAs were enriched within EVs, indicating a potentially greater role in inter-cellular transfer. The distinct compartmentalization aligns with findings that miRNAs and YRNAs exhibit a propensity for release, while lncRNAs and circRNAs are preferentially retained, expanding on recent studies highlighting miRNA sequence motifs governing their release or retention.^62^

Finally, we assessed responses to DAPT of four classes of platelet-derived plasma ncRNAs in the PACMAN-AMI trial,^25,26^ offering a cohort of 265 AMI patients with longitudinal follow-up at 24 h, 4 weeks, and 52 weeks. This was the first time that circRNAs and lncRNAs were assessed after AMI. Notably, circRNAs and lncRNAs showed similar responsiveness to DAPT as miRNAs and YRNAs. Although most effects of DAPT were achieved by 4 weeks, platelet ncRNAs decreased even further by 52 weeks. The relatively higher platelet ncRNA levels at 4 weeks vs. 52 weeks may reflect the inflammatory response in the post-acute phase of AMI, linked to increased cardiovascular risk. This effect is not captured by aggregometry.^26^ While most clinical studies use platelet count and mean platelet volume to assess platelets, ncRNA measurements could provide additional insight into platelet reactivity and serve as companion diagnostics. Conventional platelet function measurements require immediate processing of fresh blood samples, while ncRNAs are stable and can be measured in frozen plasma.

Beyond large clinical trials that have explored conventional *ex vivo* platelet function tests to guide anti-platelet therapy,^35–39^ our study—examining platelet aggregation, ncRNA release and intra-platelet ncRNA levels—is, to the best of our knowledge, the largest of its kind. However, we emphasize that correlations cannot imply causality, necessitating further mechanistic studies. Additionally, we cannot rule out that ncRNA compartmentalization and release tendency, based on samples from healthy donors, may differ in older individuals and/or patients.

Given the pivotal role of platelets in diseases and the responsiveness of plasma ncRNAs to anti-platelet therapies,^12^ understanding the dynamics of protein and ncRNA secretion by platelets is crucial. For example, sub-clinical platelet activation may contribute to the residual inflammatory risk associated with atherosclerotic cardiovascular diseases.^63,64^ Future studies should explore whether combining protein and ncRNA markers into comprehensive platelet reactivity signatures could enhance treatment guidance, considering their distinct compartmentalization and carriers.

Translational perspective Conventional *ex vivo* platelet aggregometry is rarely used clinically to guide anti-platelet therapies. Circulating platelet-derived ncRNAs have been reported as candidate biomarkers for platelet reactivity. In a large study comparing aggregometry with ncRNA measurements in platelets and their releasate, we show that platelet ncRNA release is pathway-specific and differs in terms of compartmentalization and release tendency. Additionally, we observe that inflammation associates with exhausted platelet responses *ex vivo*, potentially due to sub-clinical platelet activation *in vivo*. Post-myocardial infarction, platelet ncRNA levels were lowest after 52 weeks, although most of the DAPT effect was already achieved by 4 weeks.

## Supplementary Material

cvaf100_Supplementary_Data

## Data Availability

The data underlying this article are available in the article and in its online supplementary material. RNA-Seq data were deposited to the GEO repository with the number: GSE240195.

## STROBE and CONSORT guidelines

The STROBE checklist can be found in Supplementary material online, *Figure S13*. The CONSORT checklist can be found in Supplementary material online, *Figure S14*, while the CONSORT flow chart can be found in Supplementary material online, *Figure S3*.

## Pre-registered clinical trial number

The ClinicalTrials.gov Identifier of the PACMAN-AMI trial is: NCT03067844.
