# Supplementary material for: Platelets and inflammation—insights from platelet non-coding RNA content and release in the Bruneck study and the PACMAN-AMI trial
Source: Cardiovasc Res. 2025 Jun 3;121(9):1392–406. doi: 10.1093/cvr/cvaf100 (PMC12352307; doi:10.1093/cvr/cvaf100)
Supplement: cvaf100_Supplementary_Data [file cvaf100_supplementary_data.zip › Supplementary_Material_Online_untracked.pdf]

SUPPLEMENTARY MATERIAL

Platelets and Inflammation - Insights from Platelet Non-coding RNA Content and Release in the Bruneck Study and the PACMAN-AMI Trial

Clemens Gutmann[1,2,*], Temo Barwari[2,*], Christian Schulte[2,4,5,*], Konstantinos Theofilatos[2], Bhawana Singh[3], Kaloyan Takov[3], Gonca Suna[6], Melissa V Chan[7], Paul C. Armstrong[7], Christian Cassel[3], Yasushi Ueki[8], Jonas D. Häner[8], Peter Santer[9], Peter Willeit[10], Christian Hengstenberg[1], Lorenz Räber[8], Stefan Kiechl[11,12], Johann Willeit[11], Timothy D Warner[7], Manuel Mayr[1,3]

[1]Division of Cardiology, Medical University of Vienna, Vienna, Austria; [2]King's British Heart Foundation Centre, King's College London, London, United Kingdom; [3]National Heart and Lung Institute, Imperial College London, London, United Kingdom; [4]Department of Cardiology, University Heart & Vascular Center Hamburg, University Medical Center Hamburg-Eppendorf, Hamburg, Germany; [5]German Centre of Cardiovascular Research (DZHK), Partner Site Hamburg, Hamburg, Germany; [6]Department of Cardiology, University Heart Center Zürich, Zürich, Switzerland; [7]The Blizard Institute, Barts and The London School of Medicine and Dentistry, Queen Mary University of London, London, United Kingdom; [8]Department of Cardiology, Bern University Hospital, University of Bern, Bern, Switzerland; [9]Department of Laboratory Medicine, Bruneck Hospital, Bruneck, Italy; [10]Clinical Epidemiology Team, Medical University of Innsbruck, Innsbruck, Austria; [11]Department of Neurology, Medical University Innsbruck, Innsbruck, Austria; [12]VASCage – Centre on Clinical Stroke Research, Innsbruck, Austria.

Content included:

- Supplementary Methods
- Supplementary Tables S1-6
- Supplementary Figures S1-14
- Supplementary References

Supplementary Methods

Sample collection in the Bruneck Study

The Bruneck Study is a longitudinal study originally started in 1990, where a sex- and age-stratified random sample of 1,000 inhabitants of Bruneck (Italy) was included. In the 2015 follow-up investigation, blood of all 338 surviving Bruneck Study participants was collected after an overnight fast and 12h smoking abstinence. PRP was generated by centrifugation at 200g for 15min without brake, carefully aspirating only the uppermost part of the plasma supernatant to prevent any disturbance or aspiration of the buffy coat. 2µg/mL PGI_2 (Tocris Bioscience, cat. no. 2989) was then added to an aliquot of PRP to prevent platelet activation. PPP (*i.e.* supernatants) and platelets (*i.e.* pellets) were then obtained by a second centrifugation step at 2,200g for 10min. PRP was used for light transmission aggregometry (LTA) measurements with different platelet agonists (**Figure 1A, Supplementary material online, *Table S1***). For LTA we used an 8-channel Bio/Data PAP-8E machine, enabling us to measure all 8 agonist-treated samples of the same patient simultaneously. Final aggregation (%) was the LTA readout used for analyses as described previously.[1] Briefly, 180µL of PRP was added to eight glass cuvettes containing a magnetic stir bar. One additional glass cuvette was filled with 180µL of PPP and 20µL of PBS to serve as a negative control. All samples were then incubated at 37°C for 2min, before different platelet agonists were added to each PRP-containing cuvette. Light transmission was then continuously measured at 37°C while stirring for 5min. Aspirin use was determined using an established LTA threshold (final aggregation < 20% in response to arachidonic acid) that is superior to self-report in elderly people, as described previously.[1] Immediately afterwards, 25µL of a diclofenac/heparin mixture (final concentration: 10U/mL for heparin, 1mM for diclofenac) was added to all PRP-containing cuvettes to stop aggregation and to prevent the formation of fibrin clots. We chose 1mM diclofenac because at this concentration cyclooxygenases are completely inhibited, thereby preventing thromboxane A2 generation and any additional aggregation.[2] After LTA measurements had been completed, platelet releasates (*i.e.* supernatants) were obtained by centrifugation

at 2,200g for 10min. Platelet releasates, PPP and platelet pellets were stored at -80°C. To evaluate leukocyte contamination in our protocol, we quantified leukocytes in healthy donor PPP and PRP using the "low white blood cell mode" of a Sysmex XN-350 cell counter. Leukocytes were undetectable in PPP, whereas PRP - used for generating both platelet releasates and platelet pellets - contained 20-30 leukocytes per microliter, with a platelet count of 300,000 per microliter. The study complied with the Declaration of Helsinki and was approved by the local ethics committees of Bolzano and Verona. All study subjects provided written informed consent.[1]

Platelet lncRNA screening by RNA-Seq

To identify lncRNAs in platelets, RNA-Seq (CD Genomics, New York 11967, USA) was performed on platelet pellets collected from four healthy volunteers. For isolation of total RNA, the miRNeasy Mini kit (Qiagen, cat. no. 217004) was used according to the manufacturer's protocol. RNA quality control was performed using Qubit and agarose gel to detect the quantity of sample and using Agilent Bioanalyzer (industry standard) to determine the RNA Integrity Number (RIN). The initial input of RNA was 1.5 to 2µg. Ribosomal RNA depletion was performed using the Ribozero HMR kit (Illumina). The rRNA-depleted RNA was purified by 2x RNAClean XP beads (Beckman Coulter) and eluted in 45µL of nuclease-free water. 5µL of purified rRNA-depleted RNA was mixed with 4µL of NEBNext First Strand Synthesis Reaction Buffer and 1µL of random primers. The reaction was incubated at 94°C for 12min for fragmentation. Subsequently, to perform first strand cDNA synthesis, 8µL of NEBNext Strand Specificity Reagent and 2µL of NEBNext First Strand Synthesis Enzyme Mix was added to the reaction and the reaction was incubated at 25°C for 10 mins, 42°C for 30 mins, 70°C for 15 mins. Second strand reaction was then performed by adding 8µL of NEBNext Second Strand Synthesis Reaction Buffer with dUTP Mix (10X), 4µL of NEBNext Second Strand Synthesis Enzyme Mix, and 48µL of nuclease-free water. The reaction was incubated at 16°C for 1h. The cDNA was purified by 1.8x SPRIselect Beads (Beckman Coulter) and eluted in 50µL of nuclease-free water. Subsequently, endprep reaction was performed by adding 7µL of NEBNext Ultra II End Prep Reaction Buffer and 3µL of NEBNext Ultra II End Prep Enzyme

Mix into 50µL purified cDNA. Endprep reaction was incubated at 20°C for 30 mins and 65°C for 20 mins. The adaptor ligation reaction was then performed by adding 1µL of NEBNext Ligation Enhancer, 30µL of NEBNext Ultra II Ligation Master Mix, and 2.5µL of NEBNext Adaptor, diluted to 0.5µM in Adaptor Dilution Buffer. The mix was incubated at 20°C for 15min. 3µL of USER Enzymer (New England Biolabs) was then added to the ligation product and the reaction was incubated at 37°C for 15min. The ligated product was purified by SPRIselect Beads (Beckman Coulter) and eluted in 15µL of nuclease-free water. PCR was carried out by adding 25µL of NEBNext Ultra II Q5 Master Mix, 5µL of i5 Primer, and 5µL of i7 Primer into 15µL of purified ligated product. PCR was performed at 98°C for 30s, 15 cycles of 98°C for 10s and 65°C for 75s, and a final extension at 65°C for 5min. The final library was then purified by SPRIselect Beads.

RNA-Seq data analysis was performed using the Galaxy web server. Adapters were filtered using the Trimmomatic tool, quality control was done using the FastQC tool, mapping was performed using the HISAT2 tool against the hg19 human genome and quantification was performed using the HTSeq-count tool and lncRNAs were selected using the Biomart database annotation. The relative expressions of lncRNAs were calculated using the Fragments Per Kilobase of transcript per Million mapped reads (FPPKM) normalization. Platelet lncRNAs were ranked according to abundance (**Supplementary material online, *Table S2***). In addition, lncRNAs with the gene ontology term "platelet" were selected to prioritize lncRNA candidates associated with platelets (**Supplementary material online, *Table S3***). The RNA-Seq data reported in this study were deposited to the Gene Expression Omnibus (GEO) repository with the number: GSE240195.

Platelet circRNA validation and selection

To identify platelet circRNAs, we used a literature-based approach. Ten platelet circRNAs were selected from Alhasan et al.[3] and Preußer et al.,[4] who used RNA-Seq to screen for platelet circRNAs and had already performed RNase R validation of the targets we have chosen. To further confirm circularity, we performed our own validation using RNase R treatment of RNA isolated from platelet pellets and plasma of four

healthy volunteers as described previously.[5] Briefly, 1.1µL of RNase R master mix (Epicentre, Ref. no. RNR07250, 2U/µL RNase R) was combined with 8.9µL of RNA isolation products and incubated at 37°C for 15min in a Veriti Thermal Cycler (Applied Biosystems). The mRNA of PF4 was included as a non-circular positive control. After RNA isolation all samples were subject to four different treatment conditions before RT-qPCR: no treatment, RNase R treatment only, RNase R combined with heparinase treatment and heparinase treatment only. Heparinase treatment was included to mimic the conditions of the samples of the Bruneck Study. For qPCR measurements of circRNAs, primers with annealing sites at exon-exon backsplice junctions were used to specifically detect circular transcripts (see methods section "Quantitative polymerase chain reaction"). Lists of all primers and the target selection criteria can be found in **Supplementary material online, *Tables S4-6***.

As shown in **Supplementary material online, *Figure S2***, all 10 platelet circRNAs were detected by RT-qPCR in platelet pellets and plasma from four healthy volunteers and showed greater RNase R resistance than the positive control PF4 mRNA, confirming the data by Alhasan et al.[3] and Preußer et al.[4] We therefore chose to measure all 10 platelet circRNAs and their linear isoforms in platelet pellets of the Bruneck Study. Moreover, we chose 3 circRNAs (circUBXN7, circSMARCA5, circGSE1) for additional measurements in PPP and platelet releasates of the Bruneck Study because they emerged as promising biomarker candidates in the studies by Alhasan et al.[3] and Preußer et al.[4]: Alhasan et al.[3] found that platelet circRNAs are more stable than their linear isoforms, with the intraplatelet linear and circular isoforms of UBXN7 and SMARCA5 showing the largest divergence in abundance as platelets become older.[3] Preußer et al.[4] found that circRNAs are released from platelets via extracellular vesicles, with circGSE1 being the circRNA that is most strongly released into small extracellular vesicles.[4] Moreover, circSMARCA5 has been assessed and validated by both Alhasan et al.[3] and Preußer et al.[4]

Sample collection and platelet aggregation measurements in the PACMAN-AMI Trial

To evaluate responses of different RNA classes measured in plasma, as well as conventional aggregometry, to short-term and long-term DAPT in patients with AMI, we used the PACMAN-AMI trial. The PACMAN-

AMI trial is a randomised, double-blind, placebo controlled trial which investigates the effect of the PCSK9 inhibitor alirocumab on top of optimal medical treatment including rosuvastatin 20mg/day on coronary atheroma using serial multi-modality intracoronary imaging for nonintervened vessels in patients with AMI.[6,7] Inclusion and exclusion criteria can be found in the study protocol (NCT03067844) and in the flow chart in **Supplementary material online, *Figure S3***. The PACMAN-AMI trial complied with the Declaration of Helsinki and was approved by the institutional ethics committee. All patients provided written informed consent. Blood samples from 265 patients were collected from the antecubital vein 24 hours after hospital admission and percutaneous coronary intervention due to AMI (n = 85), and at week 4 (n = 265) and week 52 thereafter (n = 265). Samples taken at baseline (n = 265), *i.e.* immediately upon hospital admission and before alirocumab or placebo administration, were omitted to avoid comparability issues, because they were collected from the arterial sheath and not the antecubital vein. The first 2 to 4 mL of blood were discarded to avoid spontaneous platelet activation, and samples were collected in 8.2 mL citrate tubes (3.2% sodium citrate) and processed to plasma within 1 hour after blood drawing through centrifugation at 3,136g for 7min.[6,7] Platelet aggregation was measured with the VerifyNow aspirin assay and the VerifyNow $P2Y_{12}$ assay (Accumetrics Corporation, San Diego, California, Unit- ed States), as described previously.[7] Plasma was frozen at -80°C.

RNA isolation and heparinase treatment

For the isolation of total RNA, the miRNeasy Mini kit (Qiagen, cat. no. 217004) was used according to the manufacturer's protocol with some modifications.[8,9] To samples from Bruneck participants or samples from healthy volunteers used during lncRNA and circRNA validations (100µL plasma, 100µL platelet releasates, platelet pellets), 500 µL of Qiazol was added and incubated for 5min. To samples used in ultracentrifugation, size exclusion chromatography or degradation experiments (200µL each), 1,000µL of Qiazol was added and incubated for 5min. Next, 200 µL of the spiking mix was added and again incubated for 5min. This spiking mix was prepared by combining 194.75 µL Qiazol reagent with 1.25 µL MS2 carrier

RNA (Roche, cat. no. 10165948001) and 4 µL of diluted *Cel-miR-39-3p* (Qiagen, cat. no. 219600; reconstituted in 100µl dH_2O [stock solution] and further diluted 1:4000 in dH_2O [working solution]). The remaining RNA isolation steps were performed according to the manufacturer's recommendations and the RNA was eluted in 35 µL of DEPC treated, RNase-free H_2O. All RNA samples from Bruneck participants were treated with heparinase to overcome the confounding effects of heparin on qPCR measurements as described previously.[8,9] Briefly, 1.25µL heparinase I from Flavobacterium heparinum (Sigma, cat.no 9025-39-2) was combined with 3.5µl of heparinase buffer (pH 7.5), 0.25µL of RNase inhibitor (Thermofisher, Ribo Lock 40U/µL) and 5µL of RNA. The reaction was then carried out at 25°C for 3h in a Veriti Thermal Cycler (Applied Biosystems).

Reverse transcription (RT)

For reverse transcription of miRNA and YRNA, the miRCURY LNA RT kit (Exiqon, cat. no. 339340) was used. 7µL of the master mix were combined with 3µL of RNA. The reaction was then carried out according to the manufacturer's recommendations in a Veriti Thermal Cycler (Applied Biosystems) with the following incubation steps: 42°C for 1h, followed by 95°C for 5min.

For RT of mRNA, lncRNA and circRNA, the SuperScript VILO cDNA Synthesis Kit (Invitrogen, cat. no. 11755-250) was used. 2µL of VILO RT master mix were added to 8µL of RNA isolation product. The reaction was again carried out in a Veriti Thermal Cycler (Applied Biosystems) with the following incubation steps: 25°C for 10min, 42°C for 2h and 85°C for 5min. Samples belonging to the same patient were run on the same RT plate to minimize bias.

Quantitative polymerase chain reaction (qPCR)

For qPCR detection of miRNAs and YRNAs, the miRCURY SYBR Green qPCR kit (Exiqon, 339347) in combination with predesigned miRCURY LNA PCR Assays was used. For qPCR detection of mRNAs, lncRNAs and circRNAs, the SYBR™ Select Master Mix (Applied Biosystems) in combination with

custom-made primers manufactured by Integrated DNA Technologies was used. While primer sequences for all circRNAs[3,4] and the lncRNA LIPCAR[9] were derived from the literature, all other mRNA and lncRNA sequences were custom-designed, using exon-exon junction spanning primers, where applicable, to avoid amplification of genomic DNA. All circRNA primers have annealing sites at exon-exon backsplice junctions, to specifically detect circular transcripts. Lists of all primers and the target selection criteria can be found in **Supplementary material online, *Tables S4-6***. A Bravo Automated Liquid Handling Platform (Agilent) was used to prepare reactions with a volume of 5µL in 384-well plates. All qPCR measurements were performed on a ViiA7 Real-Time PCR System (Applied Biosystems) according to the manufacturer's protocols. Cq values above 35 cycles were considered undetectable.[9] Relative quantity (RQ) calculations were based on the $2^{-\Delta\Delta Cq}$ method. For normalization (ΔCq), exogenous *Cel-miR-39-3p* was used in PPP and platelet releasates, while the global average of all measured RNAs was used in platelet pellets. For $\Delta\Delta$Cq, a calibrator sample was used. For PPP and platelet releasates from Bruneck participants, the same calibrator sample consisting of a pool of 90 RNA samples (10 participants each contributing 8 releasate samples and 1 PPP sample) was measured on each 384-well plate to prevent batch effects. As an additional measure to prevent batch effects, all PPP and releasate samples of a participant were placed onto the same 384-well plate (10 participants with 8 releasate samples and 1 PPP sample each per plate). For qPCR measurements of platelet pellets from Bruneck participants, the calibrator consisted of an RNA pool from 20 participants. For all other qPCR measurements, the calibrator consisted of an RNA pool from all samples.

Enzyme-linked immunosorbent assays (ELISA)

PF4 protein levels were quantified in PPP and platelet releasates of the Bruneck Study using the Human CXCL4/PF4 DuoSet ELISA (R&D Systems, cat. no. DY795) and the DuoSet Ancillary Reagent Kit 2 (R&D Systems, cat. no. DY008) according to the manufacturer's instructions. A Tecan Infinite 200 Pro plate reader (Tecan Group Ltd.) was used to measure absorbance at 450 nm using a reference wavelength of 570 nm. Results were calculated from a four-parameter logistic fit.

Dot blots

For dot blots, 2μL of sample were applied onto a nitrocellulose membrane (Amersham Protran Premium 0.45μm NC, cat. no. GE10600002) and left to dry for 30min. Membranes were then stained with Ponceau S solution for 1-2min followed by a washing step with PBS. Membranes were then blocked using PBS-T (0.1% Tween) containing fat-free milk powder (5%) on a shaker for 60min and then placed into PBS-T for 5min. Membranes were then incubated in 50mL centrifugation tubes with primary antibodies diluted in PBS-T with 5% BSA and 0.02% sodium azide on a tube roller overnight at 4°C. The following primary antibodies were used: CD9 1μg/mL (BD, cat. no. 555370), GP1BA 1:1000 (Abcam, cat. no. ab210407), P-selectin 1:500 (Invitrogen, cat. no. 701257), PF4 0.5μg/mL (R&D, cat. no. MAB7952), APOB 1:1000 (Invitrogen, cat. no. MA5-14671). Membranes were then washed three times in PBS-T for 10min each on a shaker before 50mL secondary antibody solutions were applied for 60min at 50rpm shaking within black incubation boxes. The following secondary antibodies were used: IRDye 680RD donkey anti-mouse IgG (Immunogen, cat. no. 926-68072), IRDye 800CW donkey anti-rabbit IgG (Immunogen, cat. no. 926-32213). Thereafter, membranes were washed three times in PBS-T for 10min each on a shaker before fluorescence was detected on a LI-COR Odyssey CLx instrument.

Plasma-free platelet releasate generation

To assess the compartmentalization of platelet-derived ncRNAs, we removed plasma ncRNAs from other sources by generating plasma-free platelet releasate. Blood from healthy volunteers was drawn into sodium citrate syringes after an overnight fast. . Ethical approval for blood collections from healthy volunteers was granted from the King’s College London Ethics Committee (reference: RESCM-18/19-3676). PRP was generated by centrifugation at 200g for 15min without brake, carefully aspirating only the uppermost part of the plasma supernatant to prevent any disturbance or aspiration of the buffy coat. 2μg/mL PGI_2 (Tocris Bioscience, cat. no. 2989) and 0.02U/mL apyrase (Sigma, cat. no. A7646) were then added to an aliquot of PRP to prevent platelet activation. PRP was then centrifuged at 1,000g for 10min and the supernatant discarded. Pellets were then washed and carefully resuspended in modified Tyrode’s HEPES buffer (134

mM NaCl, 20 mM HEPES, 12 mM $NaHCO_3$, 5 mM glucose, 2.9 mM KCl, 0.34 mM Na_2HPO_4, 1mM $MgCl_2$) at pH 7.4 supplemented with 2µg/mL PGI_2 and 0.02U/mL apyrase. Several aliquots were then centrifuged again at 1,000g for 10min. Supernatants were discarded and pellets were either lysed with Qiazol or carefully resuspended in modified Tyrode's HEPES buffer supplemented with 2µg/mL PGI_2 and allowed to rest for 30min. For platelet activation, collagen (10µg/mL, Takeda, cat. no. 1130630), fibrinogen (0.25mg/mL, Sigma, cat. no. F4883) and $CaCl_2$ (2 mM) were added, and the platelet resuspension was shaken at 200rpm at 37°C for 15min. PGI_2 (2µg/mL) and diclofenac (1mM, Merck, cat. no. S0765000) were then added, and the samples centrifuged at 1,200g for 10min, followed by a second centrifugation of the supernatant at 2,200g for 10min. Platelet pellets and plasma-free platelet releasates were stored at -80°C.

Ultracentrifugation

To assess the compartmentalization of ncRNAs released from platelets, plasma-free platelet releasate samples were diluted with PBS to a volume of 1,450µL and centrifuged at 10,000g for 40min at 4°C. Pellets (large EVs) were resuspended in 1,450µL PBS and kept on ice. Supernatants were diluted with PBS to a volume of 1,450µL and centrifuged at 100,000g for 90min at 4°C, using a Beckman Coulter Optima Max ultracentrifuge with TLA-55 rotor [k-Factor 89.5] and 9.5×38mm Beckman Coulter Microcentrifuge Polypropylene Tubes. Supernatants (EV-depleted) were diluted with PBS to a volume of 1,450µL PBS and pellets (small EVs) were resuspended in 1,450µL PBS. Samples containing large EVs, small EVs and EV-depleted supernatants were then concentrated from a volume of 1,450µL down to 250µL, using 3kDa Amicon Ultra-2 Centrifugal Filter Units (Millipore, cat. no. UFC200324) in a single centrifugation at 3,000g for 30min at 4°C. Samples were then stored at -80°C. Successful separation of small EVs, large EVs and EV-depleted supernatant was confirmed by dot blot analysis. Ultracentrifugation was performed on 4 sample pools, with each pool containing samples from 6 healthy donors.

Fractionation by high-performance size-exclusion chromatography

To assess the compartmentalization of ncRNAs released from platelets, high-performance size-exclusion chromatography (SEC) of plasma-free platelet releasate samples from 9 donors was performed using a TSKgel® G5000PWXL column (hydroxylated methacrylate, particle size: 10 µm, mean pore size: 100 nm; Tosoh Bioscience, cat. no. 0008023) equipped with a TSKgel® PWXL guard column (hydroxylated methacrylate, particle size: 12 µm, mixed pore size; Tosoh Bioscience, cat. no. 0008033). To prevent RNA degradation, plasma-free platelet releasate was supplemented with 1U/µL RNaseOUT RNase inhibitor (Invitrogen, cat. no. 10777019) and SEC runs as well as all other procedures were performed at 4°C. Per SEC run, 120 µL of plasma-free platelet releasate was fractionated with PBS as a mobile phase at a flow rate of 0.6mL/min. Given that each of the 9 donors contributed 960µL plasma-free platelet releasate for SEC, 8x120µL SEC runs were performed per donor. Per SEC run, 20 fractions of 600µL each were collected in 78-second intervals and pooled as follows: pool 1 (fractions 1-3), pool 2 (fractions 4-6), pool 3 (fractions 7-8), pool 4 (fractions 9-11), pool 5 (fractions 12-16), pool 6 (fractions 17-20). The pools from all 8 SEC runs of the same donor were merged and diluted to 24mL with PBS. Samples were then concentrated down to 250µL using Vivaspin 2 (Hydrosart, 2,000 MWCO) centrifugal filter units (Sartorius, cat. no. VS02H92) at 3,000g and 4°C. Samples were then stored at -80°C. Successful fractionation was confirmed by dot blot analysis.

Protein and vesicle degradation assay

To assess the compartmentalization of ncRNAs released from platelets, we selectively degraded protein or vesicle carriers within plasma-free platelet releasate. For this purpose, 150µL of plasma-free platelet releasates from 6 donors was incubated with Triton X-100 to a final concentration of 0.1% (Sigma, cat. no. T8787), or proteinase K to a final concentration of 11.25U/mL (Sigma, cat. no. 3115887001), or modified Tyrode's HEPES buffer (negative control).[10] Reactions were carried out in a total volume of 200µL at 37°C for 45min with 75rpm shaking. Immediately thereafter, samples were lysed in 1,000µL Qiazol and stored at -80°C.

Assessment of RNA stability in plasma

To assess stability of RNAs in plasma, we collected platelet-poor plasma from 11 healthy volunteers and incubated the samples at 37°C for the following time intervals: 0h, 3h, 6h, 12h, 24h, 48h, 96h, 192h. To prevent microbial growth, blood samples were processed in a sterile tissue culture hood and supplemented with 2.5 µg/mL Amphotericin B, 100 units/mL penicillin and 100 µg/mL streptomycin. RNAs were then quantified using RT-qPCR, while PF4 protein was quantified by ELISA as described in the respective methods sections.

Imputation and Software

Imputation was performed using K nearest neighbors-based imputation with K = 5 and a 30% missing value threshold. RNA relative quantity (RQ) calculations were based on the $2^{-\Delta\Delta Cq}$ method and performed with Microsoft Excel (version 15.32). GraphPad Prism (version 9.1.1) and R programming environment (v3.6) including the packages "circlize", "ComplexHeatmap", "corrplot", "Hmisc", "lattice", "impute", "limma", "pheatmap", "tidyverse" were used for statistical analyses and to generate associated figures. Schematic diagrams were created with Biorender.com.

Supplementary Tables

Supplementary Table S1. Platelet agonists used during light transmission aggregometry (LTA).

Platelet agonist	Concentrations	Activation Pathway
Arachidonic acid (Sigma, A8798)	1 mM	Arachidonic acid
ADP (Labmedics, 3000384)	5 µM, 20µM	$P2Y_{12}$
Collagen (Takeda, 1140630)	0.4 µg/ml, 4 µg/ml, 10 µg/ml	Integrin α2β1, GPVI
Trap-6 (Bachem, H29360005)	25µM	Thrombin receptors
U46619 (Enzo, BML-PG023-0010)	10 µM	Thromboxane A2 receptor

Supplementary Table S2. Platelet RNA-Seq data for the 14 most abundant lncRNAs.

Rank	Transcript ID	Reads Sample 1	Reads Sample 2	Reads Sample 3	Reads Sample 4	Gene ID	Exon ID	Gene Name	Transcript Length	RQ value (RPKM) of Sample 1	RQ value (RPKM) of Sample 2	RQ value (RPKM) of Sample 3	RQ value (RPKM) of Sample 4	Average Expression
1	ENST00000554988	95003	133587	150782	148576	ENSG00000259001.3	ENSE00002440280	AL355075.4	638	70943.7826	83438.4746	101591.53	106318.421	90573.052
2	ENST00000575039	161922	93496	92845	80922	ENSG00000263293.2	ENSE00002643103	AC068234.2	535	144194.859	69640.533	74599.0707	69054.7187	89372.2954
3	ENST00000602361	26218	50927	55821	34646	ENSG00000269900.3	ENSE00003432004	RMRP	268	46608.2144	75724.4517	89534.7235	59019.9506	67721.835
4	ENST00000463255	57429	60093	67424	61955	ENSG00000243305.1	ENSE00001952300	AC026347.1	474	57723.2283	50520.5863	61145.5493	59673.0937	57265.6144
5	ENST00000588466	54289	55042	49856	76576	ENSG00000267112.1	ENSE00002736113	AC098848.1	567	45616.9751	38684.2325	37797.4979	61658.1021	45939.2019
6	ENST00000531241	52886	41043	43777	42070	ENSG00000255045.1	ENSE00002200533	AP000866.5	445	56621.114	36753.7726	42287.7584	43161.1528	44705.9494
7	ENST00000441746	7680	8500	8046	8579	ENSG00000227161.1	ENSE00001772568	AC092755.1	97	37721.3464	34919.6618	35656.3573	40378.0618	37168.8568
8	ENST00000643151	149941	172341	173018	218446	ENSG00000285280.2	ENSE00003828126	AL390957.1	2700	26457.8371	25435.9336	27545.8515	36936.936	29094.1396
9	ENST00000424116	29262	29373	33106	31065	ENSG00000233766.7	ENSE00001682047	AC098617.1	500	27882.4994	23409.9852	28461.9981	28364.9412	27029.856
10	ENST00000597609	21003	17916	16337	13938	ENSG00000269292.1	ENSE00002987589	AC093503.2	296	33805.4967	24119.7155	23725.1669	21497.5656	25786.9862
11	ENST00000419211	94448	78711	79186	78995	ENSG00000224746.2	ENSE00003882435	AC015987.1	1668	26977.0477	18804.5187	20407.088	21621.414	21952.5171
12	ENST00000623664	69278	200269	139768	40544	ENSG00000278996.1	ENSE00003755016	FP671120.4	2498	13212.9748	31948.0336	24051.6013	7409.94004	19155.6374
13	ENST00000578850	5183	10487	9722	9169	ENSG00000265257.5	ENSE00001881817	AP005263.1	198	12471.3572	21106.1449	21106.6401	21141.574	18956.429
14	ENST00000511893	26809	20031	23293	22048	ENSG00000251660.1	ENSE00002081580	AC007036.3	614	20802.2324	13000.4114	16307.4379	16393.8654	16625.9868

Supplementary Table S3. Platelet RNA-Seq data for lncRNAs selected by using the gene ontology term "platelet".

Rank	Transcript ID	Reads Sample 1	Reads Sample 2	Reads Sample 3	Reads Sample 4	Gene ID	Exon ID	Gene Name	Transcript Length	RQ value (RPKM) of Sample 1	RQ value (RPKM) of Sample 2	RQ value (RPKM) of Sample 3	RQ value (RPKM) of Sample 4	Average Expression
106	ENST00000668018	2477	3002	3168	2927	ENSG00000250334.6	ENSE00002036801	LINC00989	925	1275.79817	1293.27771	1472.21798	1444.64628	1371.48504
242	ENST00000664388	2224	1665	2048	1864	ENSG00000259719.6	ENSE00002567426	LINC02284	1510	701.706558	439.400076	583.017625	563.572114	571.924093
350	ENST00000451230	382	317	268	427	ENSG00000230499.1	ENSE00001761168	AC108463.2	412	441.737071	306.608989	279.618729	473.163454	375.282061
420	ENST00000521014	182	527	380	253	ENSG00000254263.1	ENSE00002099732	AC022973.4	467	185.674476	449.693461	349.780339	247.334202	308.120619
724	ENST00000428088	57	44	424	166	ENSG00000237419.1	ENSE00001591875	AL954642.1	538	50.4766216	32.5906662	338.775706	140.866053	140.677262
726	ENST00000466692	93	166	168	309	ENSG00000244459.2	ENSE00001852367	AC147067.1	579	76.5247791	114.248988	124.72669	243.646653	139.786778
813	ENST00000587754	212	181	182	204	ENSG00000267279.2	ENSE00002860442	AC090409.1	735	137.418822	98.1327735	106.441927	126.713639	117.17679
998	ENST00000533459	54	173	142	84	ENSG00000254987.1	ENSE00002201713	AP002989.1	562	45.7778239	122.668379	108.612724	68.2375625	86.3241224
1104	ENST00000657847	218	158	281	217	ENSG00000240497.3	ENSE00001950450	AC092919.1	1303	79.7094436	48.320955	92.7023157	76.0319016	74.191154
1149	ENST00000585537	41	149	113	81	ENSG00000267653.1	ENSE00002822127	AC002546.1	584	33.4478887	101.670804	83.1752807	63.3217205	70.4039235
1766	ENST00000428920	51	44	55	51	ENSG00000231039.2	ENSE00001802771	AL355303.1	680	35.7321348	25.7849683	34.7682199	34.240634	32.6314892
1941	ENST00000436786	152	198	128	121	ENSG00000234840.2	ENSE00001627949	LINC01239	2347	30.8551881	33.6182373	23.4436678	23.5370926	27.8635464
2110	ENST00000637683	92	75	57	84	ENSG00000255363.3	ENSE00003800831	LINC02757	1413	31.0201123	21.1515374	17.3404903	27.1404884	24.1631571
2221	ENST00000412811	13	52	23	16	ENSG00000231873.2	ENSE00001712642	AC099560.1	500	12.3871401	41.4434763	19.7736349	14.6093372	22.0533971
2361	ENST00000664438	23	28	20	4	ENSG00000226197.3	ENSE00001666750	AL583785.1	421	26.0281583	26.503228	20.4209799	4.33768919	19.3225139
2470	ENST00000511821	36	36	41	26	ENSG00000248223.1	ENSE00002045918	AC026785.2	867	19.7824968	16.5465037	20.3279432	13.6909879	17.5869829
3086	ENST00000665970	13	38	22	9	ENSG00000231873.2	ENSE00001715950	AC099560.1	759	8.16017131	19.9509995	12.4597573	5.41353898	11.4961168

Supplementary Table S4. Selection criteria for targets measured in the Bruneck 2015 study.

RNA	Measured in:	Selection criteria
Protein		***Literature***
PF4	PPP, Rel, PLT	Cell marker role: highly abundant in and specific to platelets. Released from platelet alpha-granules.[8]
miRNA		***Literature***
miR-122-5p	PPP, Rel, PLT	Role as non-platelet control: circulating levels specifically liver-derived.[11]
miR-21-5p	PPP, Rel, PLT	Biomarker role: circulating levels associated with platelet reactivity markers,[8] responsive to antiplatelet therapy and predominantly platelet-derived.[12] Functional role: regulates TGF-β1-release from platelets.[13]
miR-126-3p	PPP, Rel, PLT	Biomarker role: circulating levels associated with platelet reactivity markers,[8] responsive to antiplatelet therapy[12] and predominantly platelet-derived.[12] Functional role: regulates platelet activation via ADAM9 and $P2Y_{12}$.[8,14]
miR-150-5p	PPP, Rel, PLT	Biomarker role: circulating levels associated with platelet reactivity markers,[8] responsive to antiplatelet therapy and largely platelet-derived.[12]
miR-197-3p	PPP, Rel, PLT	Biomarker role: circulating levels associated with platelet reactivity markers[8] and predominantly platelet-derived.[12]
miR-223-3p	PPP, Rel, PLT	Biomarker role: circulating levels associated with platelet reactivity markers,[8] responsive to antiplatelet therapy[12] and predominantly platelet-derived.[12] Functional role: regulates platelet activation via $P2Y_{12}$.[15,16]
YRNA		***Literature***
RNY1	PPP, Rel, PLT	Biomarker role: circulating levels associated with platelet reactivity markers.[8]
RNY3	PPP, Rel, PLT	Biomarker role: circulating levels associated with platelet reactivity markers.[8]
RNY4	PPP, Rel, PLT	Biomarker role: circulating levels associated with platelet reactivity markers.[8]
RNY5	PPP, Rel, PLT	Biomarker role: circulating levels associated with platelet reactivity markers.[8]
mRNA		***Literature***
PF4	PPP, Rel, PLT	Cell marker role: highly abundant in and specific to platelets. Functional role: encodes the chemokine PF4, which is released from platelet alpha-granules.
ITGA2B	PLT	Cell marker role: highly abundant in and specific to platelets. Functional role: encodes the fibrinogen receptor on platelet membranes.
PPBP	PLT	Cell marker role: highly abundant in and specific to platelets. Functional role: encodes the precursor of the chemokine PPBP, which is released from platelet alpha-granules.
PTPRC	PLT	Cell marker role: highly abundant in leukocytes. Functional role: encodes the protein tyrosine phosphatase receptor type C.
S100A8	PLT	Cell marker role: highly abundant in neutrophils. May be transferred from neutrophils to platelets.[17] Functional role: encodes the alarmin S100A8.
S100A9	PLT	Cell marker role: highly abundant in neutrophils. May be transferred from neutrophils to platelets.[17] Functional role: encodes the alarmin S100A9.
lncRNA		***Literature & RNA-seq with RT-qPCR validation (Supplementary Figure S1)***
LIPCAR	PPP, Rel, PLT	Biomarker role (literature): circulating levels predictive of heart failure outcomes.[18] Validation: detectable by RT-qPCR in PPP, PRP and platelets of healthy volunteers.
AL3550075.4	PPP, Rel, PLT	Screening: most abundant lncRNA in platelet RNA-seq. Validation: detectable by RT-qPCR in PPP, PRP and platelets of healthy volunteers.
RMRP	PLT	Screening: third-most abundant lncRNA in platelet RNA-seq. Validation: detectable by RT-qPCR in platelets.

LINC00989	PLT	Screening: selected from RNA-seq dataset by using the gene ontology term „platelet". Validation: detectable by RT-qPCR in platelets, PRP and PPP of 4 healthy volunteers.
LINC02284	PLT	Screening: selected from RNA-seq dataset by using the gene ontology term „platelet". Validation: detectable by RT-qPCR in platelets, PRP and PPP of 4 healthy volunteers.
AL954642.1	PLT	Screening: selected from RNA-seq dataset by using the gene ontology term „platelet". Validation: detectable by RT-qPCR in platelets, PRP and PPP of 4 healthy volunteers.
AC147067.1	PLT	Screening: selected from RNA-seq dataset by using the gene ontology term „platelet". Validation: detectable by RT-qPCR in platelets, PRP and PPP of 4 healthy volunteers.
AC026785.2	PLT	Screening: selected from RNA-seq dataset by using the gene ontology term „platelet". Validation: detectable by RT-qPCR in platelets, PRP and PPP of 4 healthy volunteers.
circRNA		***Literature & RNase R validation (Supplementary Figure S2)***
GSE1	PPP, Rel, PLT	Biomarker role: enriched in and released from platelets. More strongly released into small extracellular vesicles than other investigated circRNAs.[4]
SMARCA5	PPP, Rel, PLT	Biomarker role: enriched in platelets compared to nucleated cells,[3] intraplatelet linear and circular isoforms showing larger divergence in abundance as platelets become older compared to other investigated targets,[3] released from platelets.[4]
UBXN7	PPP, Rel, PLT	Biomarker role: enriched in platelets compared to nucleated cells,[3] intraplatelet linear and circular isoforms showing larger divergence in abundance as platelets become older compared to other investigated targets,[3] released from platelets.[4]
CORO1C	PLT	Biomarker role: enriched in and released from platelets.[4]
MORC3	PLT	Biomarker role: enriched in and released from platelets.[4]
PHC3	PLT	Biomarker role: enriched in platelets compared to nucleated cells,[3] intraplatelet linear and circular isoforms showing divergence in abundance as platelets become older.[3]
Plt-circR4	PLT	Biomarker role: specific to platelets and released from platelets.[4]
PNN	PLT	Biomarker role: enriched in platelets compared to nucleated cells,[3] intraplatelet linear and circular isoforms showing divergence in abundance as platelets become older.[3]
PPA2	PLT	Biomarker role: enriched in platelets compared to nucleated cells,[3] intraplatelet linear and circular isoforms showing divergence in abundance as platelets become older.[3]
TPTEP1	PLT	Biomarker role: enriched in and released from platelets.[4]

Abbreviations: PLT: platelet pellets, PPP: platelet-poo plasma, Rel: platelet releasate.

Supplementary Table S5. List of custom-designed primers manufactured by Integrated DNA Technologies (IDT).

Primer	Forward	Reverse
PF4_mRNA	CTGCTGTTCCTGGGGTTG	GGGAGGTGGTCTTCACACAC
PPBP_mRNA	GTGATCGGGAAAGGAACCCA	AGCAGATTCATCACCTGCCA
PTPRC_mRNA	TCTTGGCATTTGGCTTTGCC	GTCAATCCAGTGGGGGAAGG
S100A8_mRNA	GCTAGAGACCGAGTGTCCTCAG	GCCCATCTTTATCACCAGAATG
S100A9_mRNA	TGGAGGACCTGGACACAAATG	TCGTCACCCTCGTGCATCTT
linGSE1	AATGTAGACGACTGGAGGCC	AACCGTCACCTGGGATATCC
circGSE1	GCCATCCTCCAGCTTTGC	GTCGCGGTGGAAAGCATC
linSMARCA5	GCTGTGTTTTGGGAAAGGTG	TCAGCTGATGAAAAGGTGCTT
circSMARCA5	TGGGCGAAAGTTCACTTAGAA	CACATGTGTTGCTCCATGTCT
linUBXN7	TCGGCAAGAACAAGAATTAAGA	TTGCATCTGGCCACACTCT
circUBXN7	TCGGCAAGAACAAGAATTAAGA	CAGGCCGTCGTCTTTTAGG
linCORO1C	CTTCATGAGAGAAAGTGTGAACC	AATGTACCCGTGCTTCAAGG
circCORO1C	ATGGGTTACATGCCCAAGAG	ACTGCTGTCACCCTTTCCAC
linMORC3	CAACTCCAAGCTTTTCTTCTCC	CCGTTTCAAGCTGTTGCTCT
circMORC3	TCTTGCCTACATCGAACGTG	ATGATCCTCGTCCCCTTCTT
linPHC3	GGAAGTGTCACACAGCAGTCA	GGGTAATACTGCCGCTGGTA
circPHC3	TCGTCATCGTCATCTTCCTG	GGGTAATACTGCCGCTGGTA
linPlt-circR4	AAGGTGTTATGTGGCCTCTG	GTGGCCACTGGAAGGATATT
circPlt-circR4	ATGCATTGGGCTACCAAGTT	GGCTGGTCACTATGCTCCTC
linPNN	GACAAAGCCCCATTTGTTTT	TGCAAATTCGATGCGTCTAC
circPNN	GACAAAGCCCCATTTGTTTT	CCTGCTTTCTCTCTTCTTCTGC
linPPA2	AGCCCTGCTCGCAGAATTAC	AGGAATGCCATTTTCCTCTTT
circPPA2	AGCTACGCTATGTGGCGAAT	AGGAATGCCATTTTCCTCTTT
linTPTEP1	GCATGAAATTTGGTGCCGTG	CTTCAGTCGCTAAGCCGAAC
circTPTEP1	GCCGACCTAATTTTCACTGACA	AGTGACATCCAGTAAGACCAGG
lncRNA_LIPCAR	TAAAGGATGCGTAGGGATGG	TTCATGATCACGCCCTCATA
lncRNA.AL355075.4*	AGCTTGGAACAGACTCACGG	AATGGGCGGAGGAGAGTAGT
lncRNA_RMRP*	AAAGTCCGCCAAGAAGCGTA	CTGCCTGCGTAACTAGAGGG
lncRNA_LINC00989*	GCTACTTGAAGGGGCACCAA	GGCGCTGAGCATTACTCTGG
lncRNA_LINC02284*	CAGCCAAAGCAGATGCAAGG	GACCCATCTGCTGAAATCCTG
lncRNA_AL954642.1*	GAAGGTGACACCAGGCAGAA	CAACAGTGATCTCCGGCTCC
lncRNA_AC147067.1*	GGACCATGAATTGTTGGAGAGGA	AAGCCCCCACTCAAATGGTT
lncRNA_AC026785.2*	GCCAAGCTAGATTCGGGTGCT	AGCGGGCTGAATGAGCAGGT

**Transcript IDs for lncRNAs selected from RNA-Seq data: lncRNA.AL355075.4: ENST00000554988, lncRNA_RMRP: ENST00000602361, lncRNA_LINC00989: ENST00000668018, lncRNA_ LINC02284: ENST00000664388, lncRNA_AL954642.1: ENST00000428088, lncRNA_AC147067.1: ENST00000466692, lncRNA_ AC026785.2: ENST00000511821.*

Supplementary Table S6. List of predesigned miRCURY LNA PCR Assays.

Primer	Manufacturer	Catalogue Nr.	GeneGlobe ID
miR-122-5p	Qiagen	339306	YP00205664
miR-21-5p	Qiagen	339306	YP00204230
miR-126-3p	Qiagen	339306	YP00204227
miR-150-5p	Qiagen	339306	YP00204660
miR-197-3p	Qiagen	339306	YP00204380
miR-223-3p	Qiagen	339306	YP00205986
RNY1	Qiagen	339317	YCP0055326
RNY3	Qiagen	339317	YCP0055329
RNY4	Qiagen	339317	YCP0055332
RNY5	Qiagen	339317	YCP0055335

Supplementary Figures

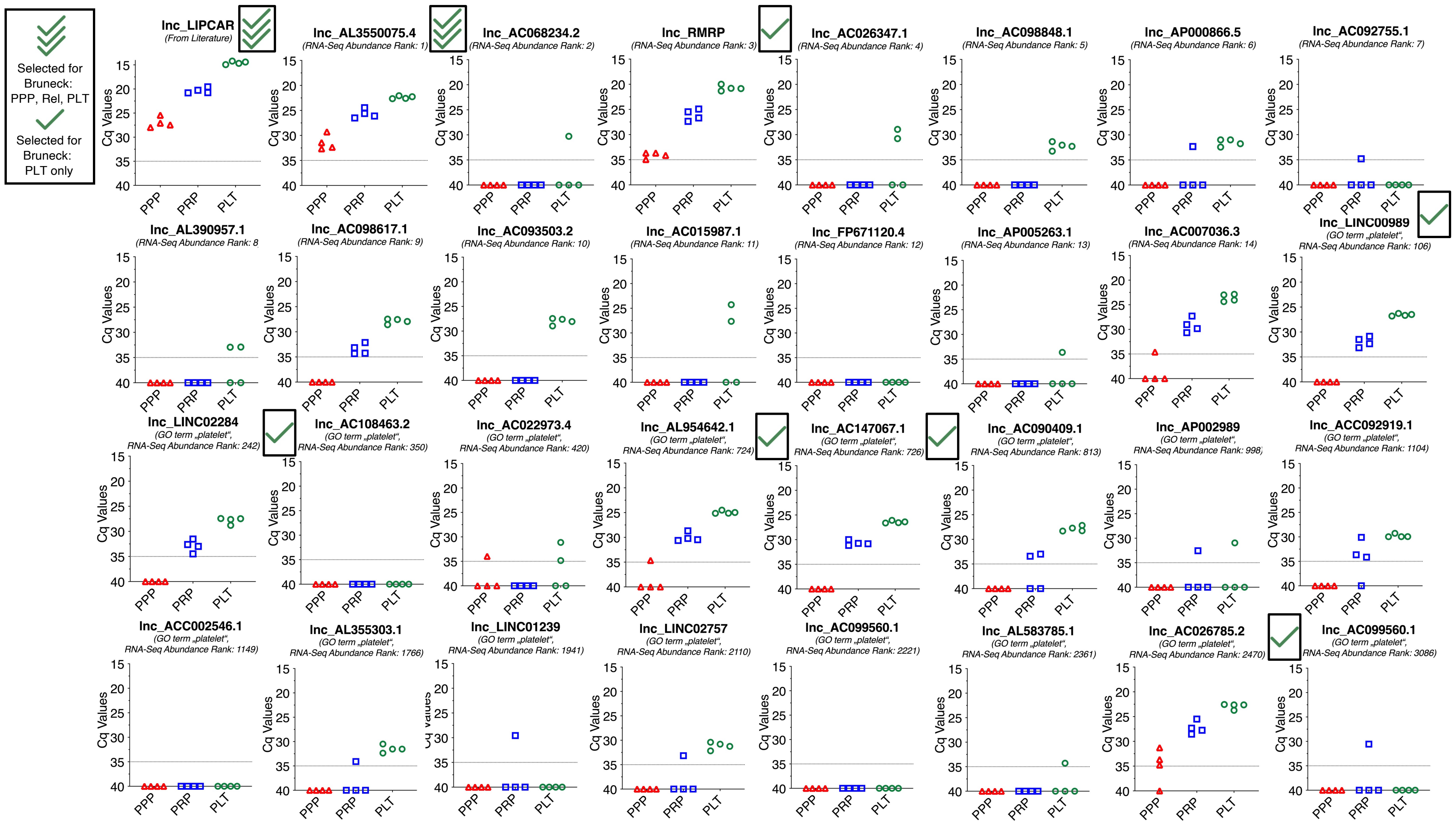

Supplementary Figure S1. RT-qPCR validation of lncRNAs. LncRNAs were selected from the literature (*i.e.* lnc_LIPCAR) or from the RNA-Seq data depending on abundancy (14 most abundant lncRNAs) or based on the gene ontology term "platelet" (17 lncRNAs). LncRNAs selected for measurement in PPP, platelet releasates (Rel) and platelet pellets (PLT) of the Bruneck Study are highlighted with three green checkmarks, whereas lncRNAs selected for measurement in platelet pellets only are highlighted with one green checkmark.

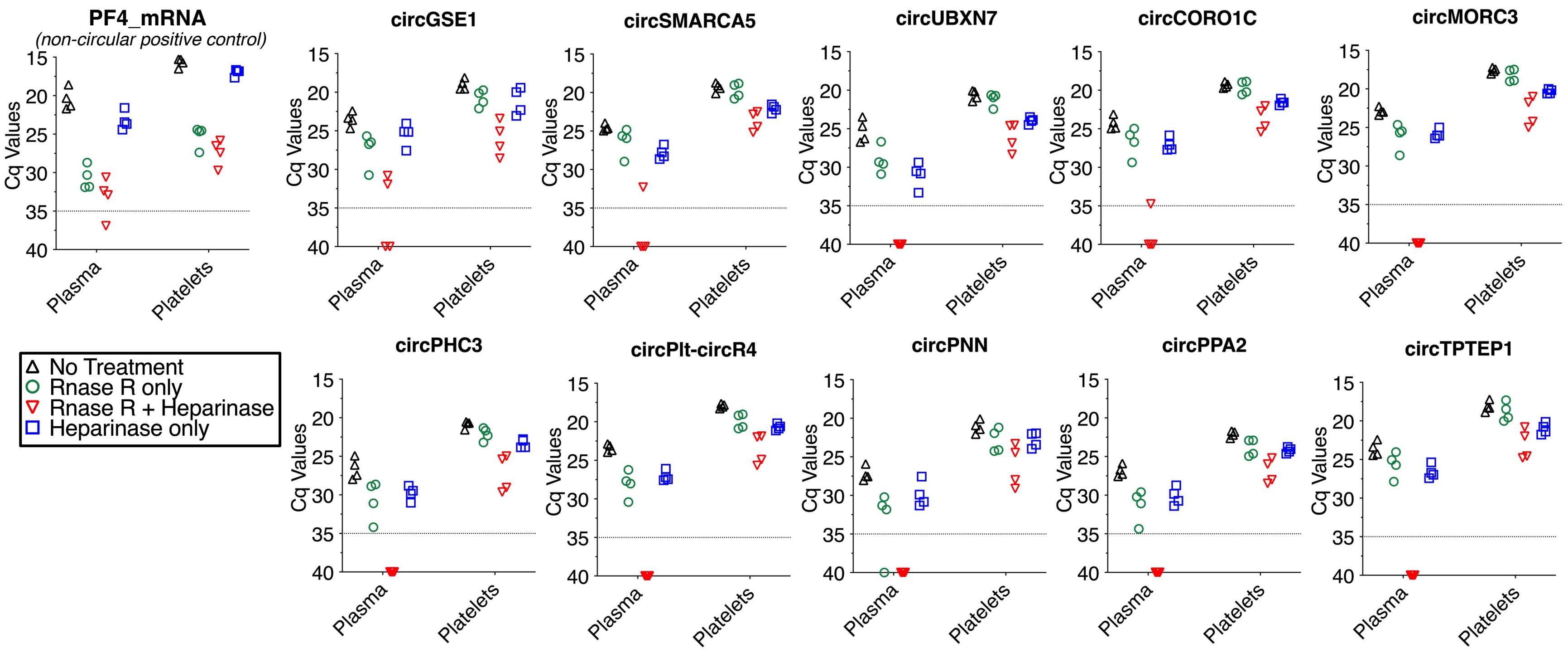

Supplementary Figure S2. Validation of circularity for circRNAs by RNase R and subsequent RT-qPCR. RNA isolated from plasma or platelet pellets from 4 healthy volunteers was split into 4 groups: no treatment (black triangles), RNase R treatment only (green circles), RNase R + heparinase treatment (red triangles) and heparinase treatment only (blue squares). PF4 mRNA was included as a non-circular positive control that is more susceptible to RNase R degradation than circRNAs.

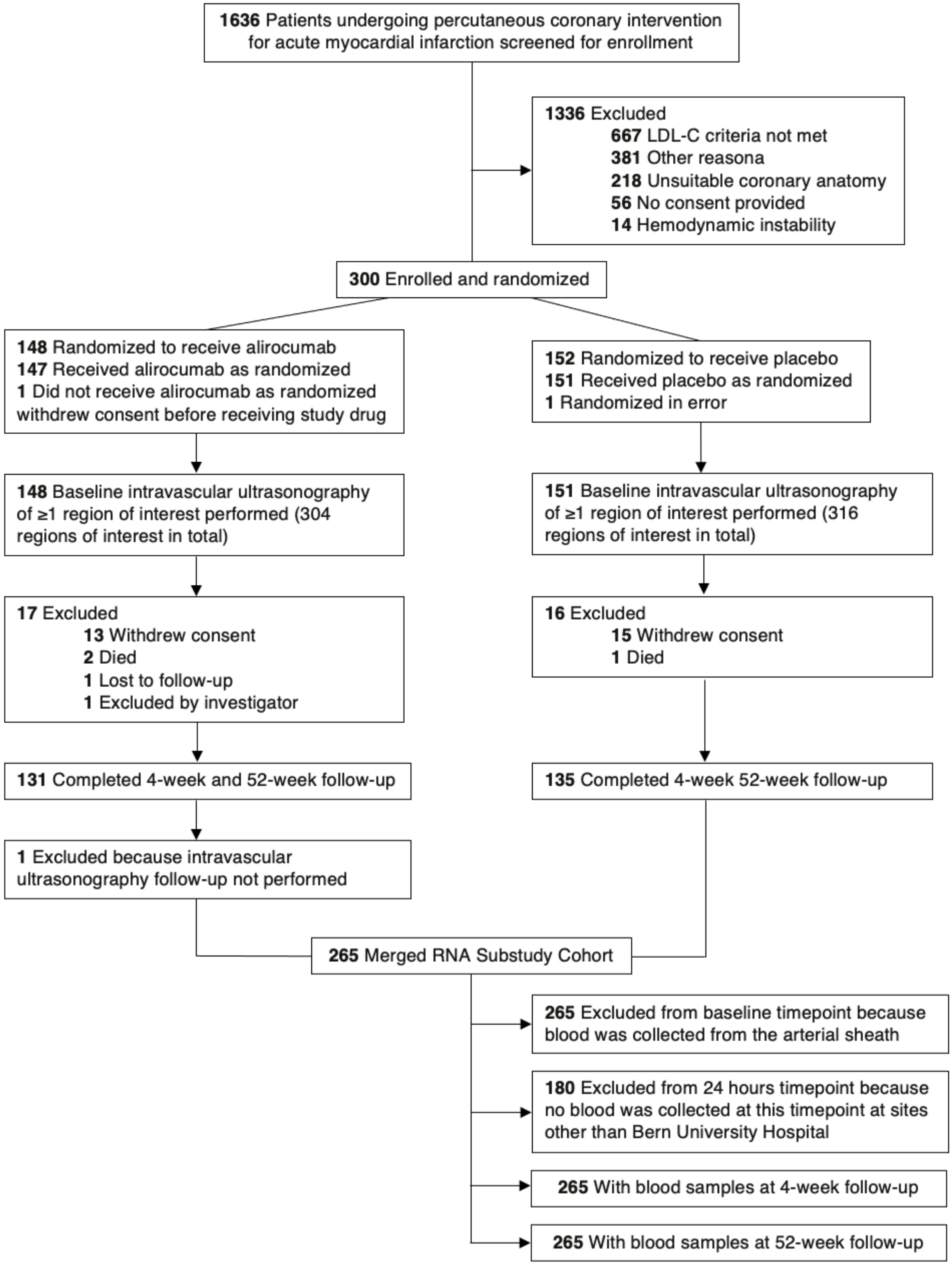

Supplementary Figure S3. CONSORT 2010 flow chart.[19] The flow chart is partly adapted from Räber et al.[6]

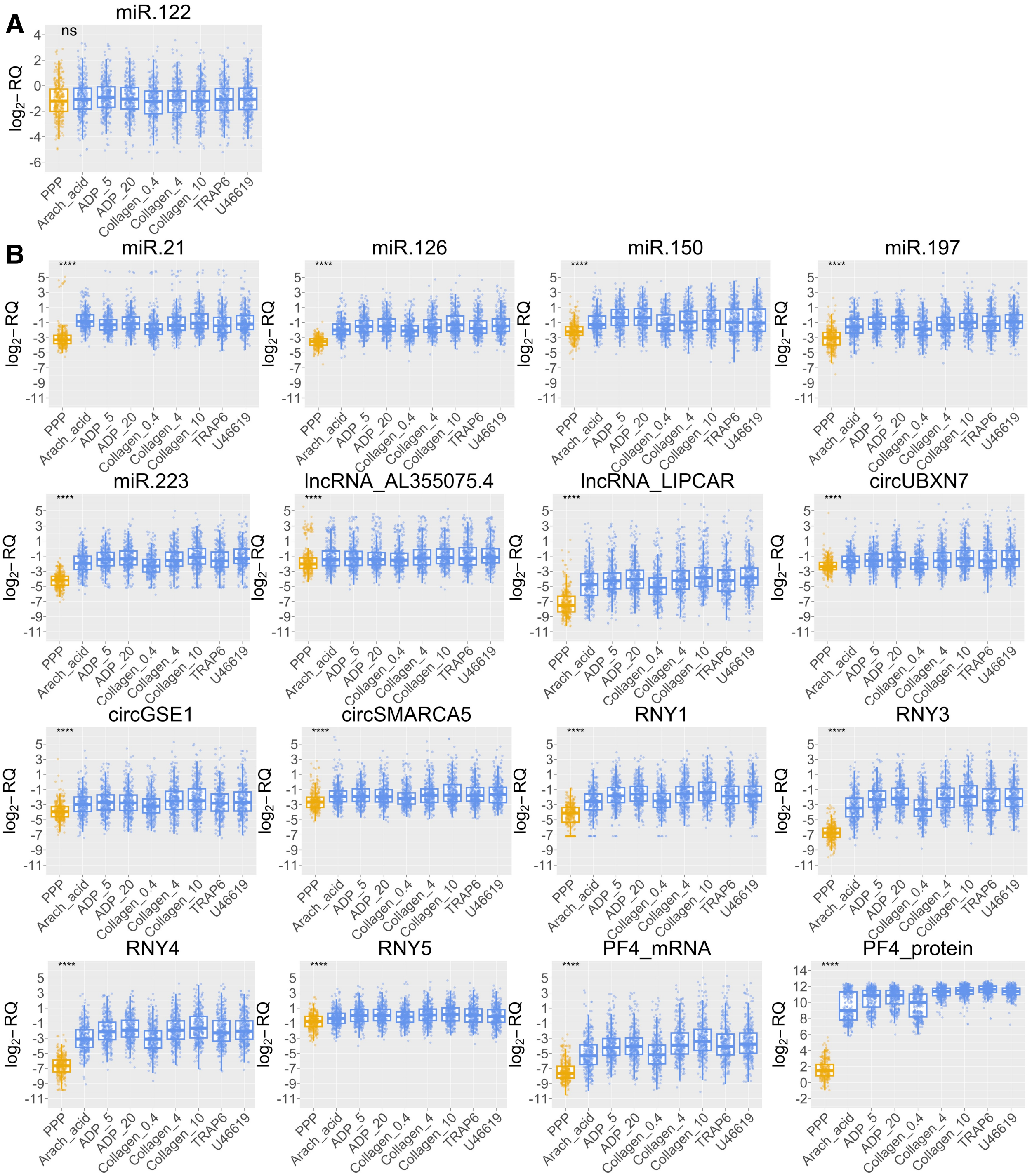

Supplementary Figure S4. (*A-B*) Box and whisker plots for hepatocyte- (*A*) and platelet-derived (*B*) targets in all 338 Bruneck participants using Kruskal-Wallis tests. The middle line represents the median, the upper and lower box borders represent the IQR and the whiskers represent 1.5 times the IQR of log_2-RQ levels.

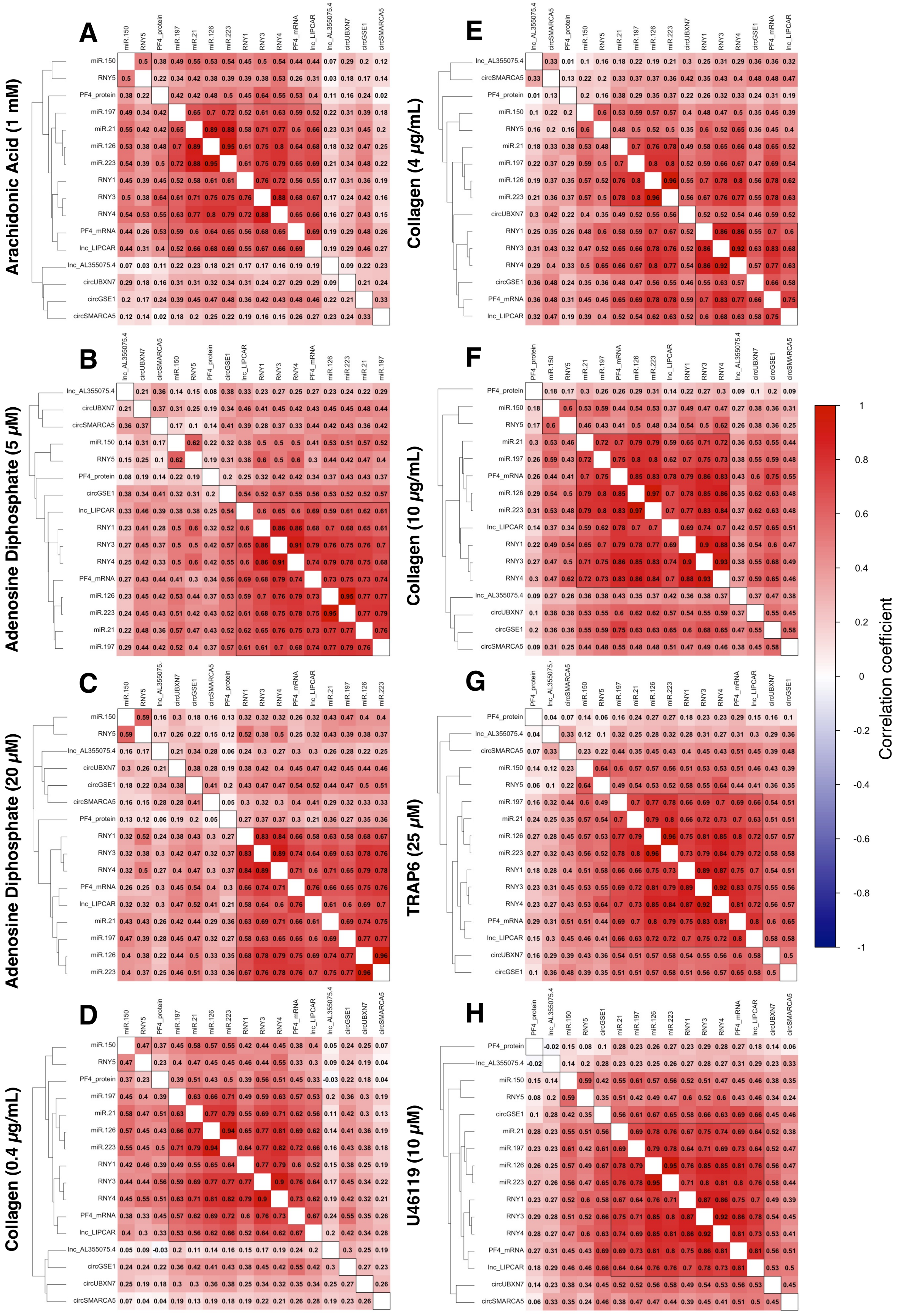
A
Arachidonic Acid (1 mM)
B
Adenosine Diphosphate (5 μM)
C
Adenosine Diphosphate (20 μM)
D
Collagen (0.4 μg/mL)
E
Collagen (4 μg/mL)
F
Collagen (10 μg/mL)
G
TRAP6 (25 μM)
H
U46119 (10 μM)
Correlation coefficient
1
0.8
0.6
0.4
0.2
0
-0.2
-0.4
-0.6
-0.8
-1

Supplementary Figure S5. Hierarchical cluster analyses on Spearman correlation heatmaps of releasate ncRNAs and PF4 protein in platelet releasates of all 338 Bruneck Study participants. Correlation coefficients are color coded and displayed within each heatmap cell. Platelet releasates were generated with 1mM arachidonic acid (*A*), 5 µM adenosine diphosphate (*B*), 20 µM adenosine diphosphate (*C*), 0.4 µg/mL collagen (*D*), 4 µg/mL collagen (*E*), 10 µg/mL collagen (*F*), TRAP6 (*G*) and U46119 (*H*).

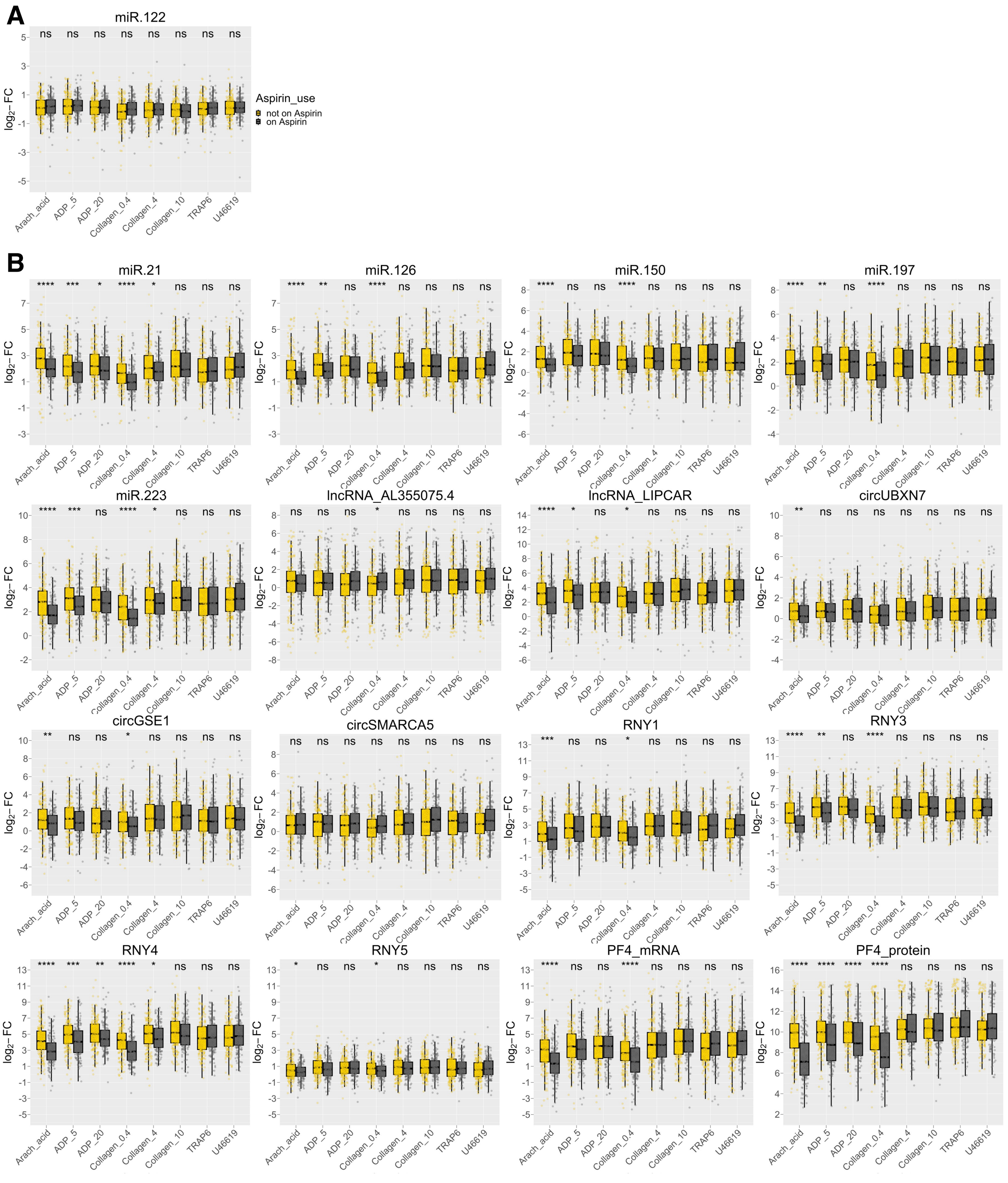

Supplementary Figure S6. (*A*-*B*), Box and whisker plots for hepatocyte- (*A*) and platelet-derived (*B*) targets comparing aspirin users (dark grey, n = 155) with non-aspirin users (n = 183) with Mann-Whitney U tests in each agonist group. The middle line represents the median, the upper and lower box borders represent the IQR and the whiskers represent 1.5 times the IQR of $\log_2$ fold change (FC) levels.

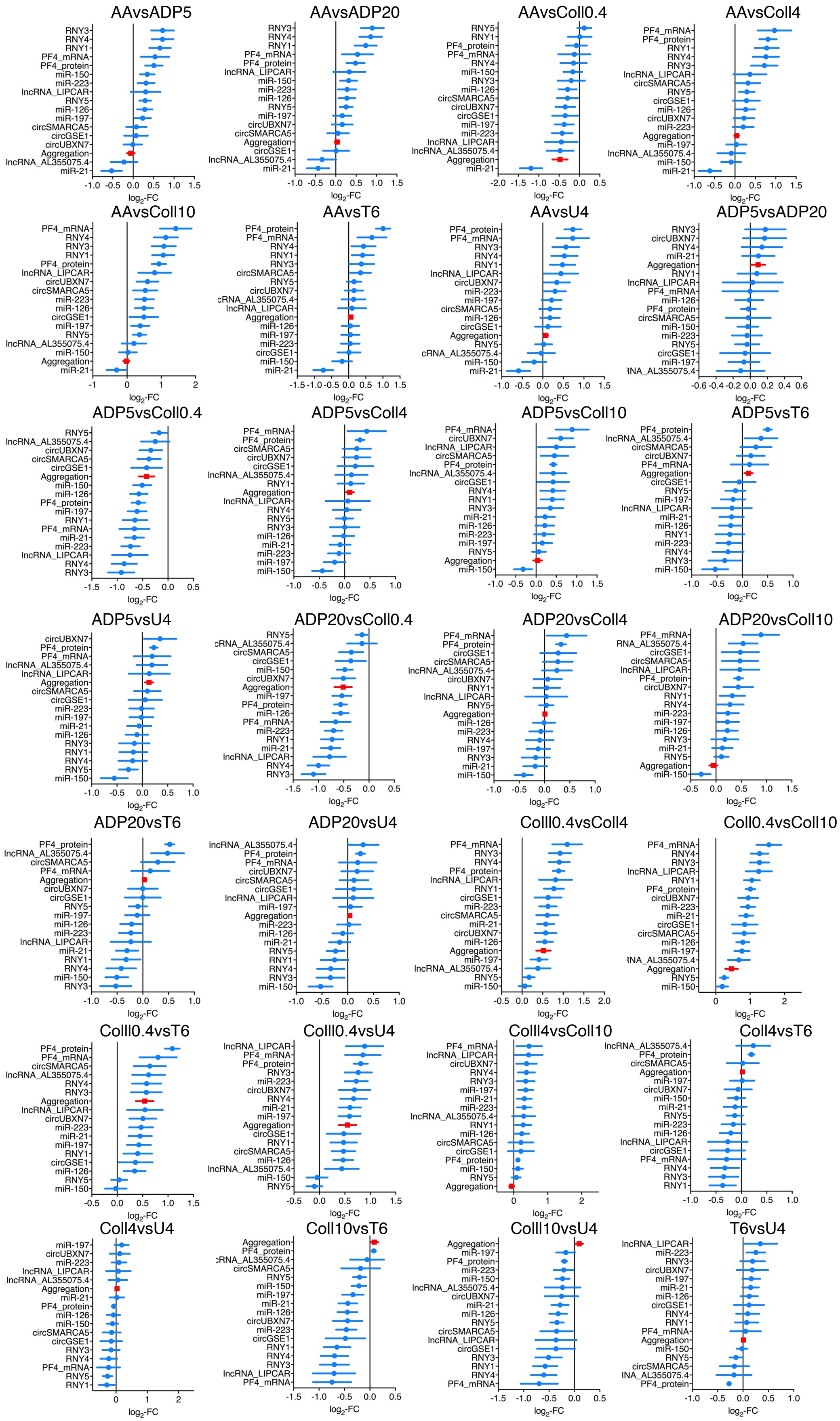
AAvsADP5
AAvsADP20
AAvsColl0.4
AAvsColl4
AAvsColl10
AAvsT6
AAvsU4
ADP5vsADP20
ADP5vsColl0.4
ADP5vsColl4
ADP5vsColl10
ADP5vsT6
ADP5vsU4
ADP20vsColl0.4
ADP20vsColl4
ADP20vsColl10
ADP20vsT6
ADP20vsU4
Colll0.4vsColl4
Coll0.4vsColl10
Colll0.4vsT6
Colll0.4vsU4
Colll4vsColl10
Coll4vsT6
Coll4vsU4
Colll10vsT6
Colll10vsU4
T6vsU4
log2-FC

Supplementary Figure S7. Logarithmised fold changes (log_2-FC) with 95% confidence intervals (CI) of RNA and protein releasate levels as well as aggregation responses between 2 representative agonists in Bruneck Study participants not on aspirin (*n* = 183).

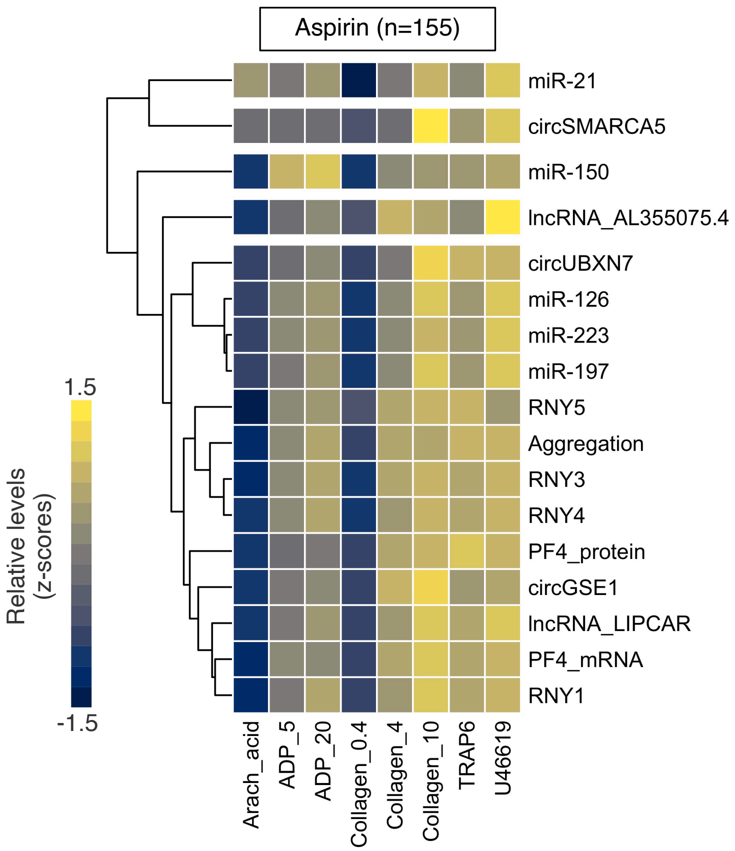

Supplementary Figure S8. Clustered heatmap displaying *ex vivo* RNA and PF4 protein release as fold changes of releasate levels to PPP levels, as well as *ex vivo* LTA aggregation responses in participants on aspirin (*n* = 155).

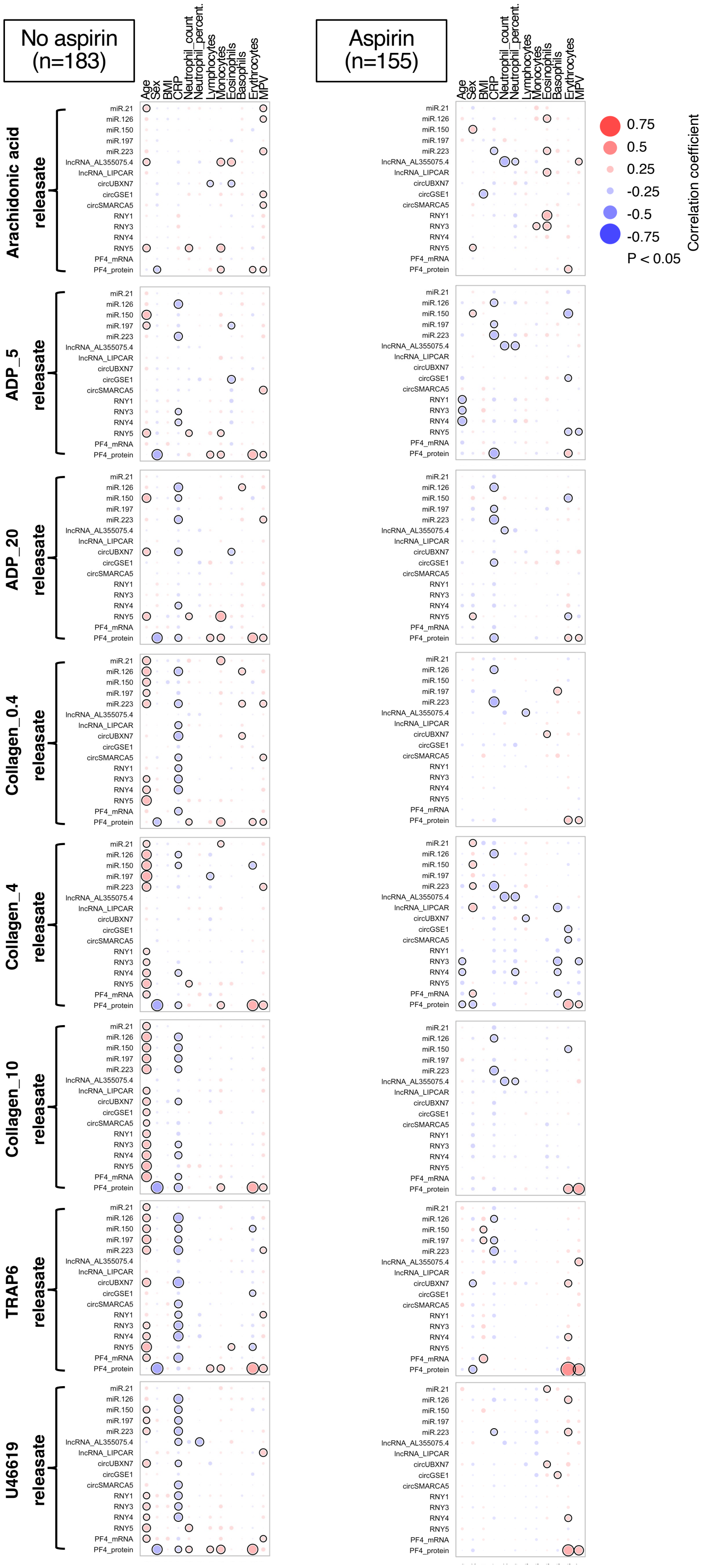

No aspirin (n=183)
Aspirin (n=155)
Age
Sex
BMI
CRP
Neutrophil_count
Neutrophil_percent.
Lymphocytes
Monocytes
Eosinophils
Basophils
Erythrocytes
MPV
miR.21
miR.126
miR.150
miR.197
miR.223
lncRNA_AL355075.4
lncRNA_LIPCAR
circUBXN7
circGSE1
circSMARCA5
RNY1
RNY3
RNY4
RNY5
PF4_mRNA
PF4_protein
Arachidonic acid releasate
ADP_5 releasate
ADP_20 releasate
Collagen_0.4 releasate
Collagen_4 releasate
Collagen_10 releasate
TRAP6 releasate
U46619 releasate
0.75
0.5
0.25
-0.25
-0.5
-0.75
P < 0.05
Correlation coefficient

Supplementary Figure S9. Heatmap displaying correlations between clinical variables and platelet releasate levels of different RNAs and PF4 protein. Analyses are stratified into participants on aspirin ($n = 155$) and participants not on aspirin ($n = 183$). Correlations between two continuous variables are based on Spearman correlation, whilst correlations between a continuous variable and a binary variable are based on Point-biserial correlation. Correlation strength is displayed by dot size and color intensity. Positive correlations are displayed in red color, whilst inverse correlations are displayed in blue color. Statistically significant ($P < 0.05$) correlations are highlighted by a black circle around the dot.

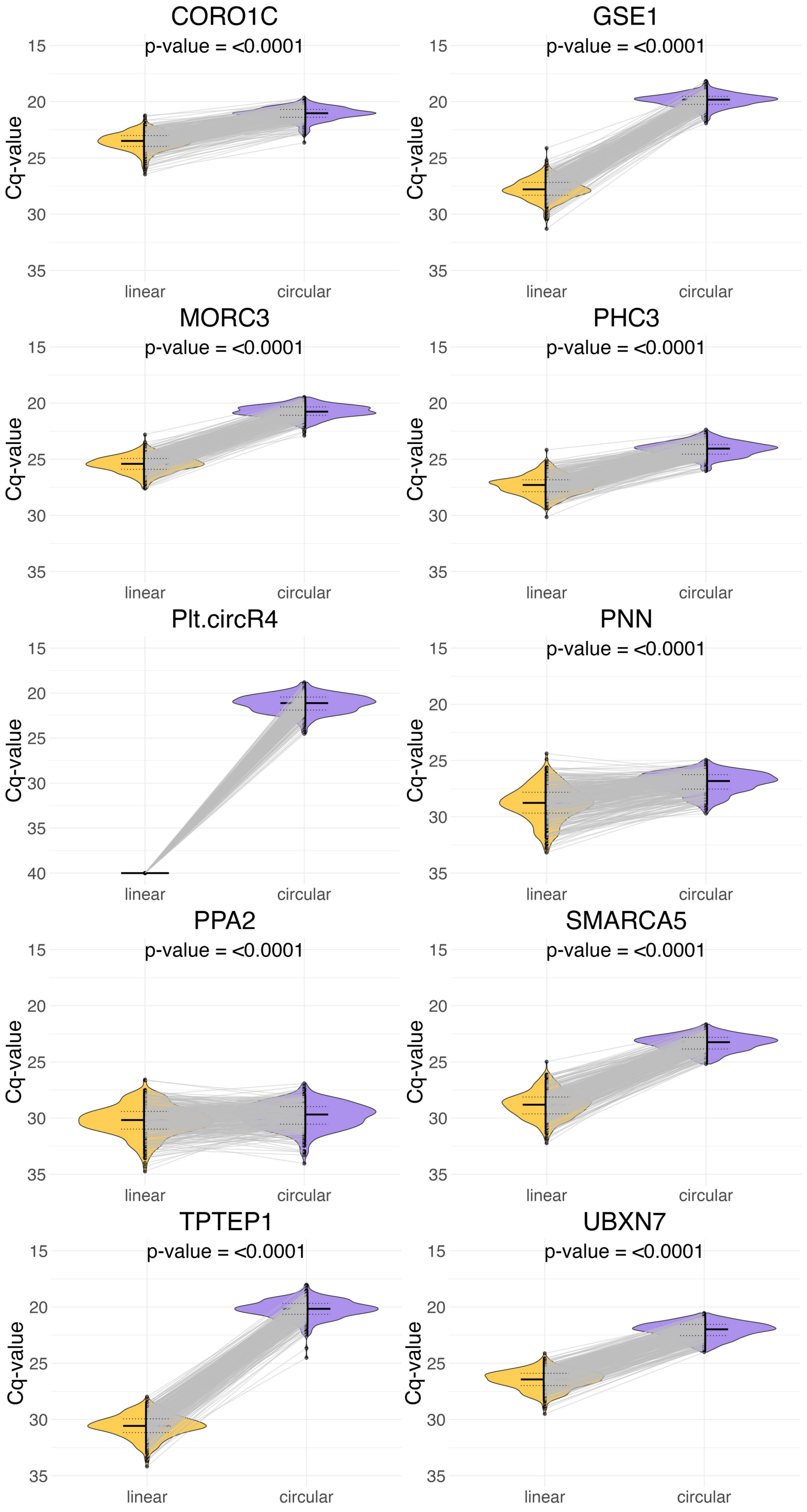
CORO1C
p-value = <0.0001
GSE1
p-value = <0.0001
MORC3
p-value = <0.0001
PHC3
p-value = <0.0001
Plt.circR4
PNN
p-value = <0.0001
PPA2
p-value = <0.0001
SMARCA5
p-value = <0.0001
TPTEP1
p-value = <0.0001
UBXN7
p-value = <0.0001
Cq-value
15
20
25
30
35
40
linear
circular

Supplementary Figure S10. Intraplatelet Cq values of circular RNAs and their linear isoforms. Violin plots display the median (middle line) and interquartile ranges (dotted lines). Paired linear and circular values of each participant (n = 338) are connected by lines. Paired Wilcoxon signed-ranks tests were used for statistical comparisons. The linear isoform of Plt.circR4 was undetectable in all samples.

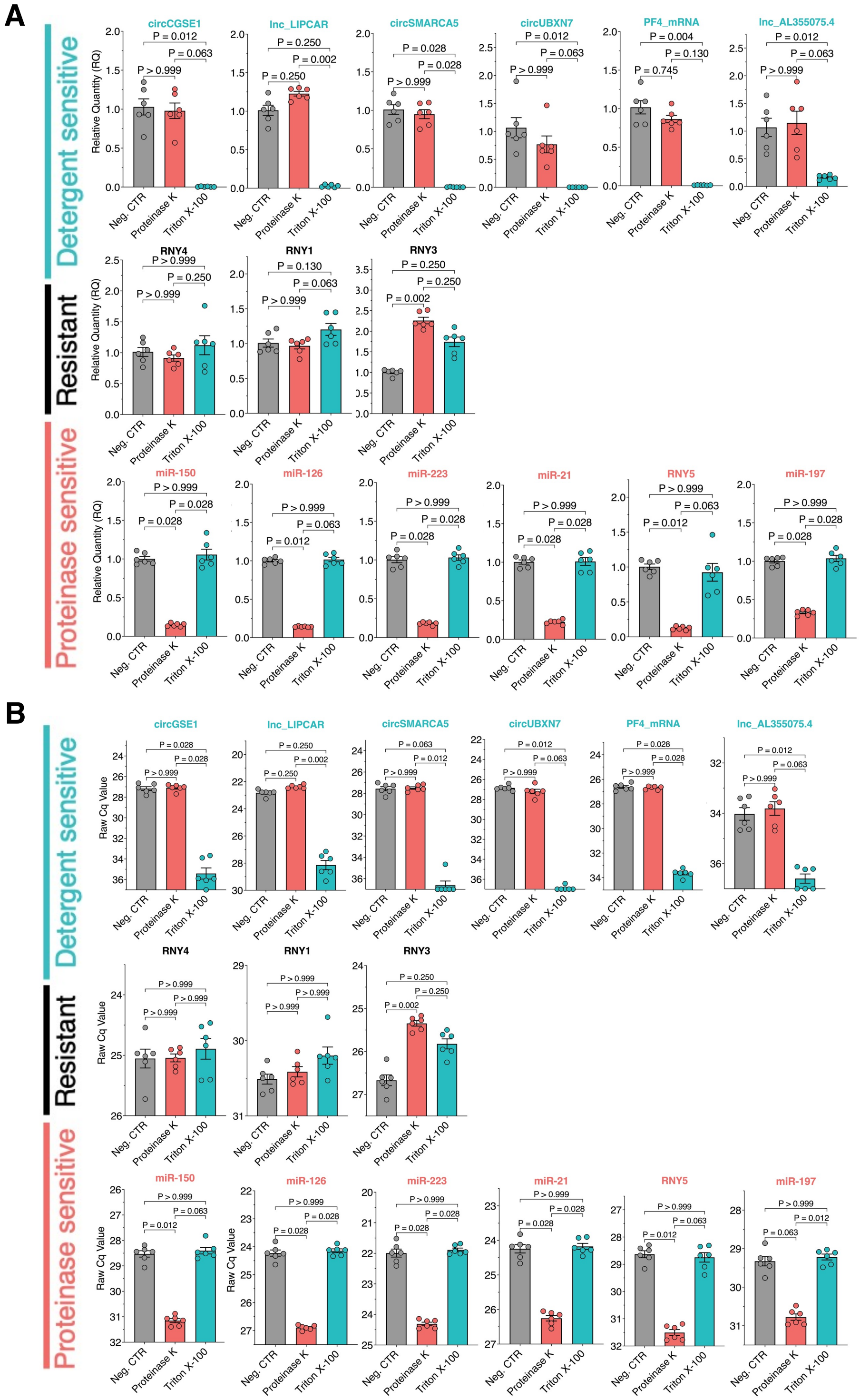

Supplementary Figure S11. Degradation assay. (*A-B*), RNA levels (*A*, relative quantities; *B*, Cq values) in samples treated with Tyrode's HEPES buffer (negative control), proteinase K or Triton-X. Bar graphs represent the mean with standard error of the mean and individual values (*n* = 6). Friedman tests with Dunn's multiple comparisons tests were used for statistical comparisons.

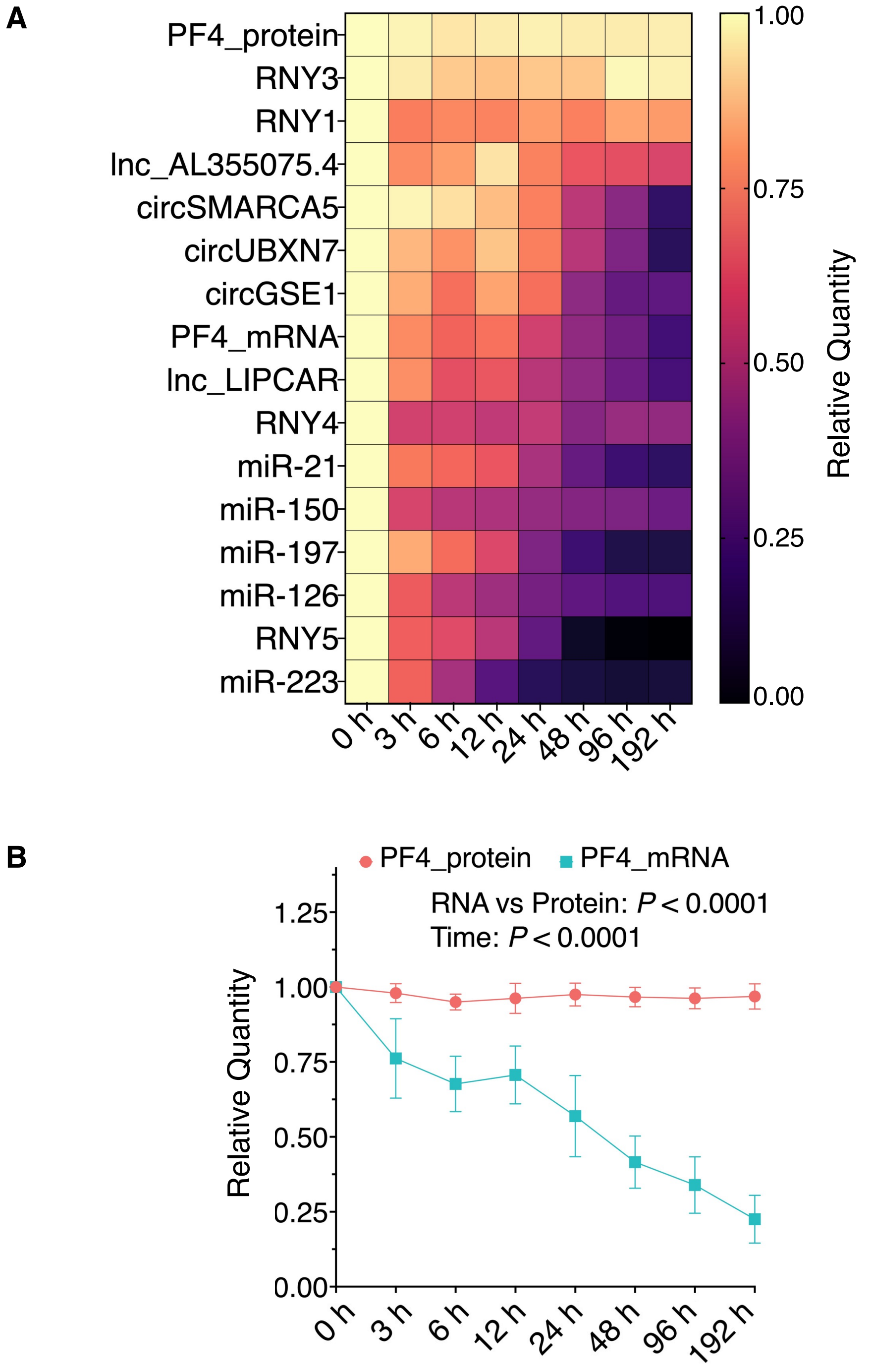

Supplementary Figure S12. Assessment of RNA stability in plasma. (*A*) Heatmap displaying mean relative RNA and protein levels in platelet-poor plasma samples from 11 healthy volunteers, incubated at 37°C for various time intervals. Measurements are ranked from highest (top) to lowest stability (bottom). (*B*) Line graph for a representative protein (PF4 protein) and a representative RNA (PF4 mRNA), displaying mean and 95% confidence intervals. Statistical comparisons were made using two-way ANOVA on n = 11 samples per group and timepoint.

STROBE Checklist.

	Item No	Recommendation	Page No
**Title and abstract**	1	(*a*) Indicate the study's design with a commonly used term in the title or the abstract<br>(*b*) Provide in the abstract an informative and balanced summary of what was done and what was found	1
**Introduction**			
Background/rationale	2	Explain the scientific background and rationale for the investigation being reported	5
Objectives	3	State specific objectives, including any prespecified hypotheses	5
**Methods**			
Study design	4	Present key elements of study design early in the paper	6
Setting	5	Describe the setting, locations, and relevant dates, including periods of recruitment, exposure, follow-up, and data collection	6
Participants	6	(*a*) Give the eligibility criteria, and the sources and methods of selection of participants. Describe methods of follow-up<br>(*b*) For matched studies, give matching criteria and number of exposed and unexposed	a: 6,<br>b: NA
Variables	7	Clearly define all outcomes, exposures, predictors, potential confounders, and effect modifiers. Give diagnostic criteria, if applicable	NA
Data sources/ measurement	8	For each variable of interest, give sources of data and details of methods of assessment (measurement). Describe comparability of assessment methods if there is more than one group	6-10
Bias	9	Describe any efforts to address potential sources of bias	10
Study size	10	Explain how the study size was arrived at	6
Quantitative variables	11	Explain how quantitative variables were handled in the analyses. If applicable, describe which groupings were chosen and why	10
Statistical methods	12	(*a*) Describe all statistical methods, including those used to control for confounding<br>(*b*) Describe any methods used to examine subgroups and interactions<br>(*c*) Explain how missing data were addressed<br>(*d*) If applicable, explain how loss to follow-up was addressed<br>(*e*) Describe any sensitivity analyses	a-c: 10,<br>d-e: NA
**Results**			
Participants	13*	(a) Report numbers of individuals at each stage of study—eg numbers potentially eligible, examined for eligibility, confirmed eligible, included in the study, completing follow-up, and analysed<br>(b) Give reasons for non-participation at each stage<br>(c) Consider use of a flow diagram	a: 11-15,<br>b-c: NA
Descriptive data	14*	(a) Give characteristics of study participants (eg demographic, clinical, social) and information on exposures and potential confounders<br>(b) Indicate number of participants with missing data for each variable of interest<br>(c) Summarise follow-up time (eg, average and total amount)	a: 34, b-<br>c: NA
Outcome data	15*	Report numbers of outcome events or summary measures over time	NA
Main results	16	(*a*) Give unadjusted estimates and, if applicable, confounder-adjusted estimates and their precision (eg, 95% confidence interval). Make clear which confounders were adjusted for and why they were included<br>(*b*) Report category boundaries when continuous variables were categorized<br>(*c*) If relevant, consider translating estimates of relative risk into absolute risk for a meaningful time period	a-b: 11-<br>15,<br>c: NA
Other analyses	17	Report other analyses done—eg analyses of subgroups and interactions, and sensitivity analyses	11-15
**Discussion**			
Key results	18	Summarise key results with reference to study objectives	18-20
Limitations	19	Discuss limitations of the study, taking into account sources of potential bias or imprecision. Discuss both direction and magnitude of any potential bias	18-20
Interpretation	20	Give a cautious overall interpretation of results considering objectives, limitations, multiplicity of analyses, results from similar studies, and other relevant evidence	18-20
Generalisability	21	Discuss the generalisability (external validity) of the study results	18-20
**Other information**			
Funding	22	Give the source of funding and the role of the funders for the present study and, if applicable, for the original study on which the present article is based	22

Supplementary Figure S13. STROBE checklist.[20]

Section/Topic	Item No	Checklist item	Reported on page No
**Title and abstract**			
	1a	Identification as a randomised trial in the title	1
	1b	Structured summary of trial design, methods, results, and conclusions (for specific guidance see CONSORT for abstracts)	3
**Introduction**			
Background and objectives	2a	Scientific background and explanation of rationale	5
	2b	Specific objectives or hypotheses	5
**Methods**			
Trial design	3a	Description of trial design (such as parallel, factorial) including allocation ratio	7-8
	3b	Important changes to methods after trial commencement (such as eligibility criteria), with reasons	7-8
Participants	4a	Eligibility criteria for participants	7-8
	4b	Settings and locations where the data were collected	7-8
Interventions	5	The interventions for each group with sufficient details to allow replication, including how and when they were actually administered	NA
Outcomes	6a	Completely defined pre-specified primary and secondary outcome measures, including how and when they were assessed	NA
	6b	Any changes to trial outcomes after the trial commenced, with reasons	NA
Sample size	7a	How sample size was determined	7-8
	7b	When applicable, explanation of any interim analyses and stopping guidelines	NA
Randomisation:			
Sequence generation	8a	Method used to generate the random allocation sequence	NA
	8b	Type of randomisation; details of any restriction (such as blocking and block size)	NA
Allocation concealment mechanism	9	Mechanism used to implement the random allocation sequence (such as sequentially numbered containers), describing any steps taken to conceal the sequence until interventions were assigned	NA
Implementation	10	Who generated the random allocation sequence, who enrolled participants, and who assigned participants to interventions	NA
Blinding	11a	If done, who was blinded after assignment to interventions (for example, participants, care providers, those assessing outcomes) and how	NA
	11b	If relevant, description of the similarity of interventions	NA
Statistical methods	12a	Statistical methods used to compare groups for primary and secondary outcomes	10
	12b	Methods for additional analyses, such as subgroup analyses and adjusted analyses	10
**Results**			
Participant flow (a diagram is strongly recommended)	13a	For each group, the numbers of participants who were randomly assigned, received intended treatment, and were analysed for the primary outcome	NA
	13b	For each group, losses and exclusions after randomisation, together with reasons	NA
Recruitment	14a	Dates defining the periods of recruitment and follow-up	17
	14b	Why the trial ended or was stopped	NA
Baseline data	15	A table showing baseline demographic and clinical characteristics for each group	7-8
Numbers analysed	16	For each group, number of participants (denominator) included in each analysis and whether the analysis was by original assigned groups	7-8
Outcomes and estimation	17a	For each primary and secondary outcome, results for each group, and the estimated effect size and its precision (such as 95% confidence interval)	NA
	17b	For binary outcomes, presentation of both absolute and relative effect sizes is recommended	NA
Ancillary analyses	18	Results of any other analyses performed, including subgroup analyses and adjusted analyses, distinguishing pre-specified from exploratory	NA
Harms	19	All important harms or unintended effects in each group (for specific guidance see CONSORT for harms)	NA
**Discussion**			
Limitations	20	Trial limitations, addressing sources of potential bias, imprecision, and, if relevant, multiplicity of analyses	20
Generalisability	21	Generalisability (external validity, applicability) of the trial findings	20
Interpretation	22	Interpretation consistent with results, balancing benefits and harms, and considering other relevant evidence	20
**Other information**			
Registration	23	Registration number and name of trial registry	22
Protocol	24	Where the full trial protocol can be accessed, if available	22
Funding	25	Sources of funding and other support (such as supply of drugs), role of funders	22

Supplementary Figure S14. CONSORT 2010 checklist.[19]

Supplementary References

1. Chan M V., Chen M-H, Barwari T, et al. Platelet Reactivity in Individuals Over 65 Years Old Is Not Modulated by Age. *Circ. Res.* 2020;127(3):394–396.
2. Warner TD, Giuliano F, Vojnovic I, et al. Nonsteroid drug selectivities for cyclo-oxygenase-1 rather than cyclo-oxygenase-2 are associated with human gastrointestinal toxicity: A full in vitro analysis. *Proc. Natl. Acad. Sci.* 1999;96(13):7563–7568.
3. Alhasan AA, Izuogu OG, Al-Balool HH, et al. Circular RNA enrichment in platelets is a signature of transcriptome degradation. *Blood*. 2016;127(9):e1–e11.
4. Preußer C, Hung L-H, Schneider T, et al. Selective release of circRNAs in platelet-derived extracellular vesicles. *J. Extracell. vesicles*. 2018;7(1):1424473.
5. Starke S, Jost I, Rossbach O, et al. Exon Circularization Requires Canonical Splice Signals. *Cell Rep.* 2015;10(1):103–111.
6. Räber L, Ueki Y, Otsuka T, et al. Effect of Alirocumab Added to High-Intensity Statin Therapy on Coronary Atherosclerosis in Patients With Acute Myocardial Infarction: The PACMAN-AMI Randomized Clinical Trial. *JAMA*. 2022;327(18):1771–1781.
7. Ueki Y, Häner JD, Losdat S, et al. Effect of Alirocumab Added to High-Intensity Statin on Platelet Reactivity and Noncoding RNAs in Patients with AMI: A Substudy of the PACMAN-AMI Trial. *Thromb. Haemost.* 2024;124(6):517–527.
8. Kaudewitz D, Skroblin P, Bender LH, et al. Association of MicroRNAs and YRNAs With Platelet Function. *Circ. Res.* 2016;118(3):420–432.
9. Schulte C, Barwari T, Joshi A, et al. Comparative Analysis of Circulating Noncoding RNAs Versus Protein Biomarkers in the Detection of Myocardial Injury. *Circ. Res.* 2019;125(3):328–340.

10. Hergenreider E, Heydt S, Tréguer K, et al. Atheroprotective communication between endothelial cells and smooth muscle cells through miRNAs. *Nat. Cell Biol.* 2012;14(3):249–56.

11. Willeit P, Skroblin P, Kiechl S, Fernández-Hernando C, Mayr M. Liver microRNAs: potential mediators and biomarkers for metabolic and cardiovascular disease? *Eur. Heart J.* 2016;37(43):3260–3266.

12. Willeit P, Zampetaki A, Dudek K, et al. Circulating MicroRNAs as Novel Biomarkers for Platelet Activation. *Circ. Res.* 2013;112(4):595–600.

13. Barwari T, Eminaga S, Mayr U, et al. Inhibition of profibrotic microRNA-21 affects platelets and their releasate. *JCI Insight*. 2018;3(21):.

14. Garcia A, Dunoyer-Geindre S, Zapilko V, et al. Functional Validation of microRNA-126-3p as a Platelet Reactivity Regulator Using Human Haematopoietic Stem Cells. *Thromb. Haemost.* 2019;119(02):254–263.

15. Landry P, Plante I, Ouellet DL, et al. Existence of a microRNA pathway in anucleate platelets. *Nat. Struct. Mol. Biol.* 2009;16(9):961–966.

16. Elgheznawy A, Shi L, Hu J, et al. Dicer Cleavage by Calpain Determines Platelet microRNA Levels and Function in Diabetes. *Circ. Res.* 2015;117(2):157–165.

17. Joshi A, Schmidt LE, Burnap SA, et al. Neutrophil-Derived Protein S100A8/A9 Alters the Platelet Proteome in Acute Myocardial Infarction and Is Associated With Changes in Platelet Reactivity. *Arterioscler. Thromb. Vasc. Biol.* 2021;ATVBAHA121317113.

18. Kumarswamy R, Bauters C, Volkmann I, et al. Circulating long noncoding RNA, LIPCAR, predicts survival in patients with heart failure. *Circ. Res.* 2014;114(10):1569–75.

19. Schulz KF, Altman DG, Moher D. CONSORT 2010 statement: updated guidelines for reporting parallel group randomised trials. *BMJ*. 2010;340(7748):698–702.

20. von Elm E, Altman DG, Egger M, et al. The Strengthening the Reporting of Observational Studies in Epidemiology (STROBE) statement: guidelines for reporting observational studies. *Lancet (London, England)*. 2007;370(9596):1453–1457.
